# Effect of Time–Temperature Modulation Combined with Residual Heating on Flavor and Texture Attributes of Steamed Perch Fish

**DOI:** 10.3390/foods15183264

**Published:** 2026-09-16

**Authors:** Yaling Yang, Lijin Wang, Feng Zhu, Huanlu Song, Yanbo Wang

**Affiliations:** 1 Laboratory of Molecular Sensory Science, School of Food and Health, Beijing Technology and Business University (BTBU), Beijing 100048, China; 18298333642@163.com (Y.Y.); songhl@th.btbu.edu.cn (H.S.); wyb1225@163.com (Y.W.); 2 Qingdao Haier Smart Technology R&D Co., Ltd., Qingdao 266100, China; zhufeng@haier.com

**Keywords:** multi-stage steaming, perch fish, flavor quality, umami compounds, aroma compounds

## Abstract

To optimize the steaming process parameters for perch fish and improve its flavor quality, this study systematically compared dual-stage steaming (low–high temperature and high–low temperature) and residual-heat steaming (dual-stage steaming followed by heat retention) with traditional single-stage steaming. Dual-stage steaming (especially 80 °C → 100 °C) produced significant treatment effects in texture and umami-related measures, while several volatile aroma compounds showed significant treatment effects (*p* < 0.05) and had higher measured concentrations under residual-heat steaming. Comparing dual-stage steaming modes (80 °C → 100 °C and 100 °C → 80 °C), the overall quality of perch fish was better when its core temperature underwent the main heating phase in the high-temperature stage. PCA comprehensive evaluation assigned the highest descriptive score (0.52) to DS 100(55)-80(75)-RH after considering texture, taste, and aroma characteristics; this ranking was not an inferential significance test. These laboratory-scale findings provide a basis for further optimization of steaming conditions for perch fish, but larger-scale validation is required before practical implementation.

## 1. Introduction

Perch fish (*Lateolabrax japonicus*) is a carnivorous freshwater fish characterized by a short growth cycle, high aquaculture profitability, and strong environmental adaptability. It possesses considerable economic and nutritional value and has become an important aquaculture species worldwide, particularly in China, Japan, and Türkiye [1,2,3]. Owing to its tender flesh, rich nutrients, and distinctive flavor [4,5,6], perch fish is highly favored by consumers, and market demand continues to increase. Common culinary methods for perch fish include steaming, baking, salting, and boiling [7,8]. Among these, steaming is considered a relatively mild thermal process, as it avoids the excessive heat damage of frying and minimizes flavor loss caused by leaching during boiling [9]. Consequently, steaming better preserves both the nutritional value and intrinsic flavor of fish [10,11] and has become a widely adopted and health-conscious cooking method for perch fish.

Traditional steaming typically involves heating perch fish with saturated steam at 100 °C, a simple and cost-effective approach in household cooking and catering practices. However, traditional steaming exhibits notable limitations in process control, with temperature and duration often determined empirically. Insufficient steaming may result in undercooking of the fish core and hinder the aromatic flavor of steamed perch fish. In contrast, excessive steaming can cause severe protein denaturation, pronounced muscle fiber contraction, and substantial moisture loss, ultimately resulting in a dry and tough texture, reduced tenderness, and diminished flavor intensity [12,13,14]. In practical household and catering operations, fish may be left in the closed steamer after active heating ends. During this residual-heat stage, energy stored in the chamber, steam, cookware, and fish continues to transfer to the fish without energy input. This carryover cooking may affect texture and flavor. However, the specific impact of such residual-heat cooking on the texture, aroma and umami taste of perch fish, particularly when integrated with a segmented steaming strategy, has rarely been systematically investigated. Nevertheless, increasing consumer expectations for flavor quality and the catering industry’s demand for consistency and standardization highlight the inadequacy of traditional single-stage steaming.

With the widespread adoption of various smart cooking appliances, such as steam ovens with programmable temperature control, segmented steaming has become increasingly feasible. Segmented steaming divides the heating process into multiple stages with different temperature settings, allowing precise regulation of heating rates and muscle structure changes. Previous studies have explored the application of segmented thermal processing in meat products. Yao et al. [15] reported that dual-stage heating of pork blocks, with preheating at 50–60 °C for 20–35 min followed by high-temperature treatment, significantly reduced shear force (from 16 N to 13.8 N) and increased moisture content (from 39.84% to 40.99–46.27%) by promoting uniform myofibrillar separation and reduced fiber gaps. Similarly, Noh et al. [16] found that segmented boiling of chicken breast (55 °C for 180 min followed by 75 °C for 8.5 min) reduced shear force from 17.35 N to 12.50 N while maintaining moisture content comparable to conventional treatment (75 °C for 30 min). These findings indicate that segmented heating can effectively improve meat tenderness without excessive moisture loss. Currently, research on segmented steaming of perch fish remains limited. Wang et al. [17] compared traditional single-stage steaming at 100 °C with segmented steaming (100 °C followed by 75 °C) on the quality of perch fish and found that segmented treatment reduced protein oxidation and increased volatile aldehydes and alcohols, enhancing the characteristic aroma of steamed perch fish. However, the systematic impact of different temperature combinations and transition points on perch fish flavor quality has not been comprehensively investigated.

Tenderness, aroma, and umami taste are important components of the sensory quality of thermally processed fish products. Liu et al. [18] observed that steaming pufferfish (*Takifugu flavidus*) for 5 min reduced hardness to 66.79% of the raw fish, while 10 min increased hardness and chewiness, indicating an optimal texture at intermediate times. Regarding aroma development, Wang et al. [19] reported that (*E*)-2-octenal was a compound unique to cooked perch fish, with significant increases in hexanal, ethyl acetate, 2-butanone, and 2-pentylfuran, suggesting that steaming enriches perch fish aroma through lipid oxidation. In terms of umami, Wang et al. [20] found that umami intensity in cured black carp (*Mylopharyngodon piceus*) peaked after 6–8 min of steaming, based on equivalent umami concentration and taste activity value analyses. Nevertheless, existing studies have mainly focused on single cooking conditions or individual quality attributes across different fish species, and systematic investigations into the coordinated changes in tenderness, aroma, and umami of perch fish under different steaming strategies remain limited [21,22].

Therefore, this study established a dual-stage steaming system with multiple temperature combinations and incorporated a residual-heat cooking process. Core temperatures of perch fish were continuously monitored to define transition points between steaming stages. Texture profile analysis, high-performance liquid chromatography (HPLC), and gas chromatography–olfactometry–mass spectrometry (GC-O-MS) were employed to comprehensively evaluate shear force, textural properties, umami-related compounds, and volatile aroma profiles of perch fish under different steaming conditions. This study aims to clarify the effects of temperature segmentation and residual-heat cooking on flavor formation in perch fish, providing theoretical insights and technical references for optimizing and standardizing steaming processes for perch fish.

## 2. Materials and Methods

### 2.1. Materials

#### 2.1.1. Perch Fish Samples

Fresh perch fish were purchased from Xiaoxiang Supermarket (Beijing, China). All fish originated from the same aquaculture production batch, and individuals of similar body weight (approximately 500 g per fish) were selected to reduce raw-material variability. Scales and viscera were removed manually, and fish were rinsed thoroughly under running water. Surface and abdominal moisture were gently removed using absorbent paper towels to minimize the influence of external water on steaming efficiency and subsequent quality analysis.

#### 2.1.2. Chemicals and Reagents

Potassium dihydrogen phosphate, disodium hydrogen phosphate, phosphoric acid, sodium hydroxide, trichloroacetic acid, sodium chloride, methanol, acetonitrile, and *n*-hexane (chromatographic grade) were all purchased from Merida Technology Co., Ltd. (Beijing, China). Adenosine 5′-monophosphate disodium salt (5′-AMP), inosine 5′-monophosphate disodium salt (5′-IMP), and guanosine 5′-monophosphate disodium salt (5′-GMP) were purchased from Yuanye Bio-Technology Co., Ltd. (Shanghai, China). o-Phthalaldehyde (OPA), 9-fluorenylmethyl chloroformate (FMOC), borate buffer solution, and the amino acid mixed standard solution (1 nmol/μL in 0.1 M HCl) were purchased from Agilent Technologies Co., Ltd. (Santa Clara, CA, USA).

### 2.2. Steaming Conditions

After preprocessing, perch fish samples were placed flat on white porcelain plates and steamed using a steam oven (Casadei, Qingdao, China). Three steaming modes were investigated comparatively: single-stage steaming, dual-stage steaming, and residual-heat steaming. Detailed processing parameters are summarized in Table 1.

A thermocouple temperature recorder (TP9000, Shenzhen Toprie Electronics Co., Shenzhen, China) was employed to continuously monitor the core temperature of the perch fish during steaming. Temperature probes were inserted into the geometric center of the dorsal muscle. For each fish, three probes were carefully positioned in the thickest part of the muscle tissue while avoiding bones and other rigid structures. The core temperature of each sample was calculated as the average value of the three measurement points. For all treatments, steaming was terminated when the core temperature reached 80 °C, which was considered sufficient to ensure complete cooking and optimal quality. For dual-stage steaming, the oven temperature was adjusted to initiate the second stage when the core temperature reached 45, 55, or 65 °C, respectively. For residual-heat steaming, heating was immediately stopped once the core temperature reached 75 °C, while the oven door remained closed. The samples were then held under residual heat within the chamber until the core temperature increased naturally to the endpoint of 80 °C.

Each steaming condition was performed in three independent heating batches (*n* = 3 experimental replicates). Within each batch, three perch fish were steamed simultaneously and shared the same thermal environment; these fish were treated as subsamples rather than independent replicates. Dorsal muscle from the three fish was pooled and homogenized to produce one batch-level sample. Thus, the independent heating batch was the experimental unit for physicochemical and flavor analyses.

### 2.3. Appearance and Sensory Evaluation

The appearance and sensory evaluation panel consisted of 10 trained assessors (5 males and 5 females, aged 23–35 years) from the Molecular Sensory Science Laboratory of Beijing Technology and Business University, each with over one year of experience in sensory evaluation. Prior to formal assessment, the panel underwent a two-week intensive training covering evaluation procedures, attribute definitions, and familiarization with reference standards.

#### 2.3.1. Training Procedures

(1) Appearance evaluation

Color was assessed against the natural creamy-white of freshly cooked perch fish, with yellowish or dark tones considered undesirable. Tissue structure, elasticity, and integrity were assessed using reference samples and the grading criteria in Table S1.

(2) Odor evaluation

Panelists’ olfactory recognition ability was calibrated using chemical reference standards, including fishy odor (nonanal, 10 μg/mL), grassy odor (hexanal, 20 μg/mL), fatty odor (octanal, 15 μg/mL), and mushroom-like odor (1-octen-3-ol, 5 μg/mL). A six-point intensity scale was applied, ranging from 0 (not perceived) to 5 (extremely strong), with intermediate levels defined as extremely weak, weak, moderate, and strong.

(3) Texture and taste evaluation

Freshly cooked perch fish samples were used for standardized assessments of tenderness (resistance when biting), moistness (juice release/lubrication), and chewiness (chews/force to disintegrate). Taste was calibrated with aqueous references: bitter (1 mmol/L caffeine) and umami (10 mmol/L monosodium glutamate).

#### 2.3.2. Formal Evaluation

All sensory tests were conducted in a dedicated sensory evaluation room under uniform artificial daylight illumination (6500 K). Before evaluation, all steamed fish samples were maintained at 40 ± 2 °C and served immediately. The presentation order of the test samples was independently randomized for each assessor.

After training, appearance, odor, texture, and taste were evaluated sequentially. For appearance assessment, intact perch fish samples processed under different steaming conditions were visually evaluated and scored for four attributes: color, tissue structure, elasticity, and integrity, according to the criteria described in Table S1. Odor evaluation followed, with panelists gently sniffing the samples and scoring the intensity of five odor attributes: cooked fish meat odor, fatty odor, grassy odor, fishy odor, and mushroom-like odor, using the six-point scale. For texture and taste evaluations, panelists placed an appropriate portion of fish flesh into the mouth and assessed tenderness, moistness, chewiness, as well as the intensity of umami and bitterness. The same intensity scale used for odor evaluation was applied to taste attributes. All samples were served on white plates and labeled with random three-digit codes. Between samples, panelists rinsed their mouths with purified warm water, waited for 1 min, and consumed a small piece of unsalted plain cracker before evaluating the next sample. The mean scores obtained from all panelists were calculated and used as the final sensory evaluation results. The protocol was approved by the Ethics Committee of Beijing Technology and Business University, with informed consent obtained.

### 2.4. Texture Analysis

#### 2.4.1. Shear Force

After steaming, dorsal muscle tissues were cut into cubes (approximately 2 cm per side). Shear force was measured using a texture analyzer (TA.XTC-18, Bosin Tech, Shanghai, China) equipped with a plastic blade. The test parameters were as follows: pre-test speed, 120 mm/min; test speed, 60 mm/min; post-test speed, 60 mm/min; compression ratio, 90%; and trigger force, 10 g. Eight cubes were randomly selected from each sample, and the mean shear force value was used as the replicate result.

#### 2.4.2. Texture Profile Analysis (TPA)

TPA was performed to evaluate the textural properties of steamed perch fish, including hardness, springiness, chewiness, cohesiveness, and resilience, using the same texture analyzer with a P/25 spherical probe (25 mm diameter). The TPA test consisted of two consecutive compression cycles to simulate mastication. The operating parameters were set as follows: pre-test speed, 3.00 mm/s; test speed, 1.00 mm/s; post-test speed, 1.00 mm/s; compression strain, 30%; trigger force, 5 g; and a 5 s interval between the two compression cycles. For each sample, eight cubes were randomly selected for measurement, and the average value was calculated as the replicate result.

### 2.5. Determination of Umami-Related Compounds in Perch Fish

#### 2.5.1. Determination of Free Amino Acids

The extraction of free amino acids was performed according to the method of Sun et al. [23] with minor modifications. Briefly, 2 g of minced dorsal muscle from steamed perch fish was homogenized with deionized water (1:3, *w*/*v*), followed by the addition of 2 mL deionized water and 2 mL of 10% (*w*/*v*) trichloroacetic acid (TCA). The homogenate was centrifuged at 10,000 rpm for 10 min at 4 °C using a refrigerated centrifuge (CR22N, Hitachi, Tokyo, Japan). The supernatant was collected, and the extraction was repeated twice under identical conditions. The combined supernatants were filtered through a 0.22 μm microporous membrane and stored at −80 °C prior to analysis.

Free amino acids were quantified using an Agilent 1260 HPLC system equipped with a diode array detector (Agilent Technologies, Palo Alto, CA, USA). Separation was performed on a Zorbax Eclipse-AAA column (150 mm × 4.6 mm, 5 µm) following pre-column derivatization with OPA-FMOC reagents. The derivatization system consisted of 0.4 mol/L borate buffer, OPA reagent, FMOC reagent, and ultrapure water. Detection was carried out at 338 nm. The column temperature was maintained at 40 °C, the flow rate was 1.0 mL/min, and the injection volume was 5 μL. Mobile phase A was 0.04 mol/L NaH_2_PO_4_ aqueous solution (pH 7.8), while mobile phase B was a mixture of methanol, acetonitrile, and water (45:45:10, *v*/*v*/*v*). The gradient elution program was as follows: 0–1.9 min, 100% A; 18.1–23.2 min, 63% A; 24–26 min, 0% A; and 27–30 min, 100% A.

Quantitative analysis was performed using an external standard method. An amino acid mixed standard was diluted with 0.1 mol/L HCl to obtain standards at concentrations of 0.125, 0.250, 0.500, 0.750, and 1.000 nmol/μL. Free amino acid contents in the samples were calculated by fitting peak areas to the corresponding calibration curves.

#### 2.5.2. Determination of Nucleotides

Nucleotides were extracted using the same procedure described in Section 2.5.1.

Nucleotide analysis was conducted using an Agilent 1200 series HPLC system equipped with a DAD (Agilent Technologies, Palo Alto, CA, USA). Separation was performed on an Agilent Eclipse XDB-C18 column (250 mm × 4.6 mm, 5 µm). Mobile phase A consisted of 0.02 mol/L KH_2_PO_4_ solution adjusted to pH 3.8 with H_3_PO_4_, while mobile phase B was 10% (*v*/*v*) methanol in water. The gradient program was as follows: 0–10 min, 100% A; 11–26 min, 0% A; and 26–27.5 min, 100% A. The injection volume was 1 μL, the detection wavelength was set at 254 nm, the column temperature was maintained at 35 °C, and the flow rate was 1.0 mL/min.

IMP, AMP, and GMP were used as external standards. Standard solutions were prepared at concentrations of 25, 50, 100, 200, and 400 mg/L to construct calibration curves. Nucleotide contents in the samples were quantified using the external standard method.

#### 2.5.3. Equivalent Umami Concentration

Equivalent Umami Concentration (EUC) is expressed as grams of monosodium glutamate (MSG) equivalent per 100 g of sample (g MSG/100 g) and calculated using the following equation [24]:

(1) $$EUC=\left(\sum a_{i}\cdot b_{i}\right)+1218\cdot \left(\sum a_{i}\cdot b_{i}\right)\cdot (\sum a_{j}\cdot b_{j})$$

where

a_i_: Content of umami-active amino acids (Glutamic acid or Aspartic acid, g/100 g);

a_j_: Content of umami-active nucleotides (5′-GMP, 5′-IMP, 5′-XMP, or 5′-AMP, g/100 g);

b_i_: Relative umami coefficient of amino acids compared with Glu (Glu = 1, Asp = 0.077);

b_j_: Relative umami coefficient of nucleotides compared with 5′-IMP (5′-IMP = 1, 5′-AMP = 0.18, 5′-GMP = 2.3, 5′-XMP = 0.61);

1218: Synergistic interaction constant between umami-active amino acids and nucleotides.

### 2.6. Determination of Aroma Compounds

#### 2.6.1. Solid-Phase Microextraction (SPME)

The extraction of volatile aroma compounds was performed according to the method of Wang et al. [25] with minor modifications. Approximately 5.00 g of steamed perch fish muscle was minced uniformly and mixed with saturated sodium chloride solution at a meat-to-solution mass ratio of 7:3. The mixture was thoroughly homogenized, and 1 μL of 2-methyl-3-heptanone (0.0816 μg/μL) was added as an internal standard. The sample was equilibrated at 70 °C for 20 min in 20 mL headspace vials. DVB/CAR/PDMS fiber (50/30 μm, Supelco, Bellefonte, PA, USA) was exposed to the headspace for 40 min to extract volatile compounds. After extraction, the fiber was immediately inserted into the GC injection port for thermal desorption at 230 °C for 5 min. Each sample was analyzed in triplicate.

#### 2.6.2. GC-O-MS Analysis Conditions

Volatile aroma compounds in steamed perch fish were analyzed using a GC-MS (7890A/7000B, Agilent, Santa Clara, CA, USA) equipped with an olfactometry detection system (Sniffer 9100; Brechbühler, Switzerland). Chromatographic separation was achieved on a DB-WAX capillary column (30 m × 0.25 mm × 0.25 μm, J & W Scientific, Folsom, CA, USA). Ultra-high purity helium was used as the carrier gas at a constant flow rate of 1.0 mL/min, and samples were injected in splitless mode. The oven temperature program was as follows: initial temperature of 40 °C held for 5 min, increased to 230 °C at a rate of 4 °C/min, and held at 230 °C for 5 min. Mass spectrometry was conducted using electron impact ionization at 70 eV, with a scan range of *m*/*z* 40–450. Each sample was analyzed in triplicate.

The GC-O analysis was performed by a sensory panel consisting of three trained assessors (one male, two females, aged 20–25 years). All assessors had at least 100 h of prior GC-O experience and were trained for an additional 20 h. During GC-O, each assessor recorded the odor description and intensity in real time using a sniffing port (Sniffer 9100). A compound was considered olfactorily detected if at least two of the three assessors perceived an odor at the same retention time. The transfer line and sniffing port were maintained at 280 °C and 200 °C, respectively, and humidified nitrogen was supplied at the sniffing port.

#### 2.6.3. Relative Odor Activity Value Analysis

The relative odor activity value (rOAV) was calculated using the following equation [26]:

(2) $$rOAV=\frac{C_{i}}{10\cdot OT_{i}}$$

where C_i_ is the concentration of compound i in the sample (ng/g), and Oti is the odor threshold of compound i in water (μg/100 g). Compounds with higher rOAVs were interpreted as making a greater relative contribution to the overall aroma profile of the sample.

### 2.7. Statistical Analysis

All experimental data were processed using Excel 2007 and are expressed as mean ± standard deviation. Statistical analyses were performed using SPSS Statistics 26 (IBM Corp., Chicago, IL, USA). One-way analysis of variance (ANOVA) was applied to evaluate significant differences among treatments, followed by Duncan’s multiple range test for post hoc comparisons. Differences were considered statistically significant at *p* < 0.05. Prior to principal component analysis (PCA), Z-score standardization was conducted on all selected variables to eliminate the influence of inconsistent units and numerical ranges of quality indicators. All figures were generated using Origin 2024 (OriginLab Corp., Northampton, MA, USA).

## 3. Results and Discussion

### 3.1. Temperature-Time Curves of Perch Fish Under Single-Stage Steaming, Dual-Stage Steaming, and Residual-Heat Steaming

**Figure 1:**
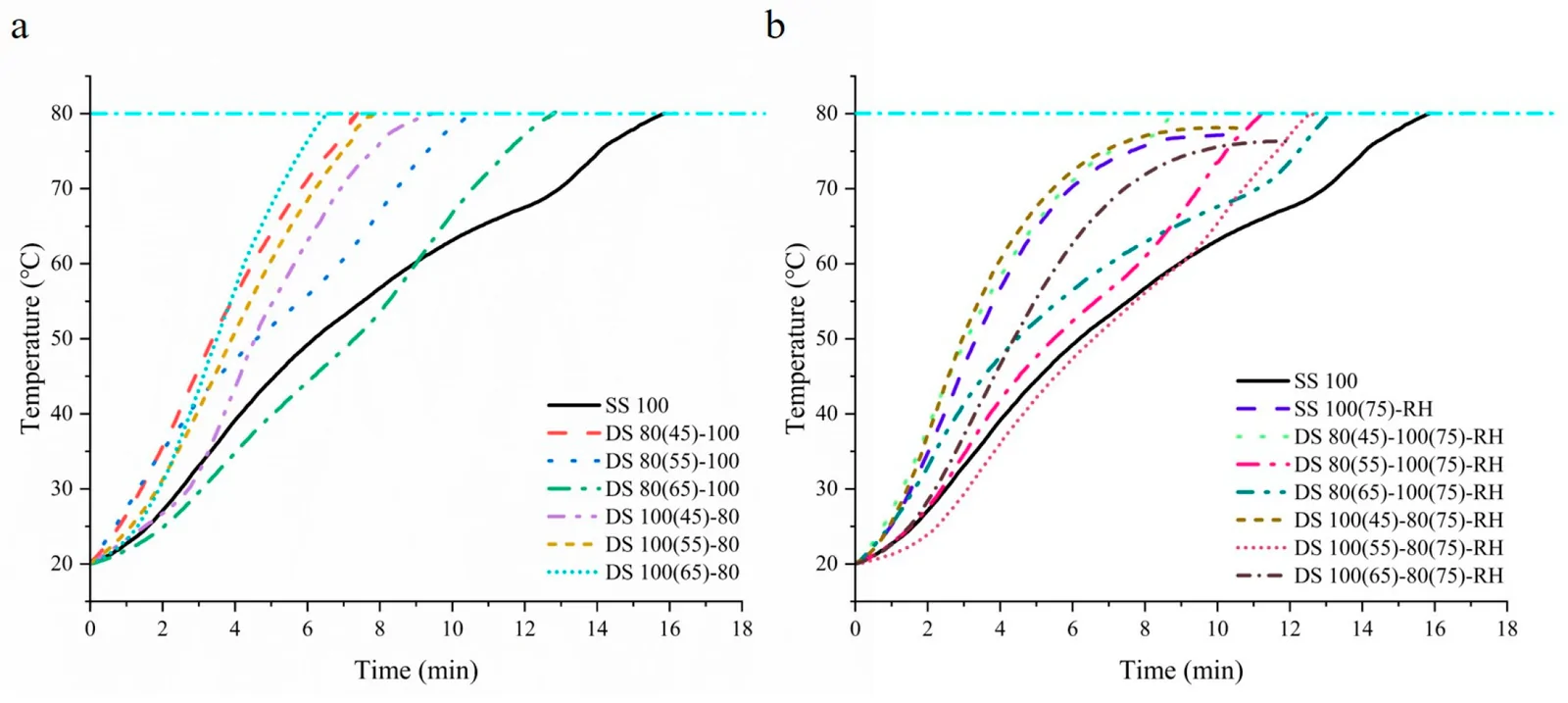
Core temperature-time curves of perch fish under different steaming modes ((**a**): Single-stage and dual-stage steaming at various transition temperatures; (**b**): Single-stage and residual-heat steaming at various transition temperatures).

During steaming, the core temperature of perch fish was continuously recorded, and the temperature–time curves are shown in Figure 1. All steaming modes exhibited a similar three-stage heating pattern: an initial slow stage due to the large temperature gradient and thermal inertia, a middle stage of accelerated heating as steam condensation enhanced heat transfer, and a final stage where the heating rate gradually stabilized. Under single-stage steaming at 100 °C (SS 100), the core temperature rose slowly at first, then increased rapidly, and finally reached 80 °C at 15.84 min. The slow initial phase resulted from the time needed for heat to penetrate from the surface to the core, while the later slowdown was likely due to protein denaturation and gel-layer formation, which increased thermal resistance.

Compared with single-stage steaming, both dual-stage steaming and residual-heat steaming substantially shortened the time required for the perch fish core to reach the target temperature. The total steaming time was reduced from 15.84 min under SS 100 to 6.54–14.76 min under these alternative modes, indicating a pronounced improvement in overall heating efficiency. For dual-stage steaming with an oven temperature sequence of 80 °C → 100 °C (Figure 1a), the total steaming time varied significantly depending on the selected core-temperature transition point. The times required to reach 80 °C were 7.38 min for DS 80(45)-100, 10.56 min for DS 80(55)-100, and 12.6 min for DS 80(65)-100, respectively. As the transition temperature increased, the total steaming time was progressively prolonged. This is because when the oven temperature was switched from 80 °C to 100 °C at a core temperature of 45 °C, the subsequent 35 °C increase (from 45 °C to 80 °C) occurred entirely under the high-temperature condition (100 °C), where heat transfer efficiency was maximized, resulting in a shorter overall steaming time. In contrast, delaying the temperature switch until the core reached 65 °C meant that most of the temperature rise occurred under the lower oven temperature of 80 °C, which reduced heat transfer efficiency and extended the total processing time. An opposite trend was observed for the 100 °C → 80 °C sequence. As the transition temperature increased, the total steaming time decreased. Specifically, DS 100(45)-80, DS 100(55)-80, and DS 100(65)-80 reached the target temperature in 9.48 min, 7.86 min, and 6.54 min. This indicates that allocating a larger proportion of the temperature rise to the high-temperature stage (100 °C) accelerates heating. For example, when the oven temperature was switched from 100 °C to 80 °C at a core temperature of 65 °C, the high-temperature stage (100 °C) had already accomplished a 45 °C rise (from 20 °C to 65 °C), while only a minor temperature rise (15 °C) was required under the lower-temperature condition (80 °C), leading to the shortest steaming duration. In contrast, an early switch at 45 °C limited the contribution of the high-temperature stage, forcing a larger temperature increase to occur under less efficient heating conditions and resulting in a longer total steaming time. When residual-heat steaming was applied using the same dual-stage strategy, heating trends remained similar but total steaming times generally increased (Figure 1b). For the 80 °C → 100 °C sequence, residual-heat steaming required 9.00–14.76 min, longer than the corresponding dual-stage steaming (6.54–10.56 min), likely due to the lower effective oven temperature and reduced temperature gradient during the residual-heat phase. For the 100 °C → 80 °C sequence, the core temperature failed to reach 80 °C, plateauing near 77 °C before declining, likely because the residual thermal energy at the second-stage 80 °C was insufficient to sustain further heat transfer.

### 3.2. Appearance and Sensory Evaluation of Perch Fish Quality Under Single-Stage Steaming, Dual-Stage Steaming and Residual-Heat Steaming

**Figure 2:**
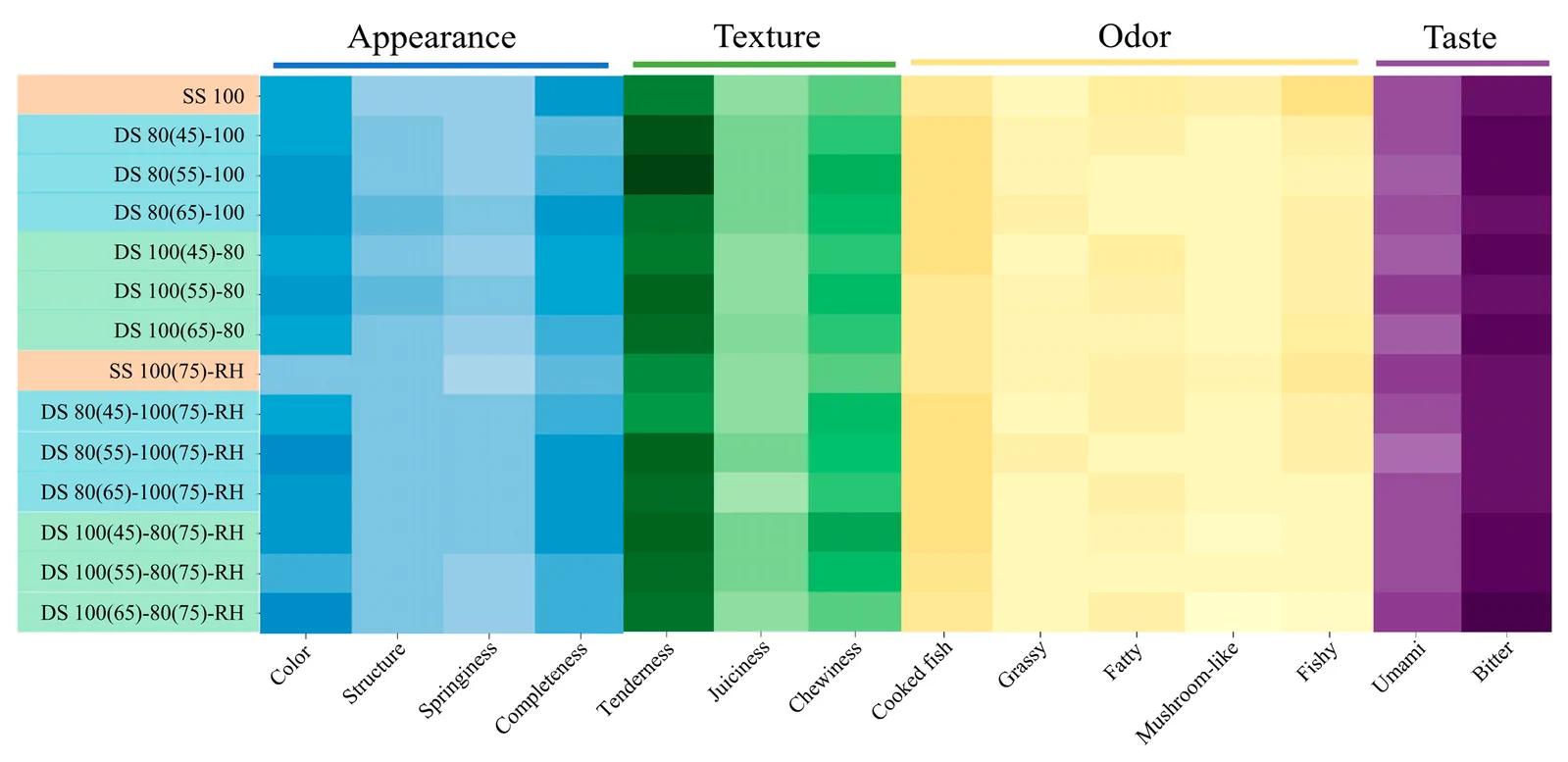
Heatmap of appearance, texture, odor, and taste scores of perch fish under different steaming modes.

Figure 2 summarizes the appearance, texture, odor, and taste scores for all steaming treatments. In terms of appearance (Figure 2 and Figure S1), the samples exhibited differences in color, tissue structure, elasticity, and integrity; however, these variations were not statistically significant (*p* > 0.05). Treatments DS 100(55)-80 and DS 100(45)-80(75)-RH had numerically higher appearance scores, but these differences should not be interpreted as significant treatment effects. Texture perception indicated comparable moisture perception, tenderness, and chewiness among the steaming conditions (*p* > 0.05). Residual-heat treatments DS 100(45)-80(75)-RH and DS 80(55)-100(75)-RH received numerically higher scores for tenderness and elasticity. DS 100(65)-80(75)-RH received lower numerical scores for moisture perception and chewiness. These nonsignificant patterns are consistent with previous reports that heat-induced changes in water status can influence perceived juiciness and chewiness [27,28]. Umami and bitterness scores did not differ significantly among treatments (*p* > 0.05; Figure 2). Therefore, the observed numerical differences in taste scores were not interpreted as significant treatment effects. With respect to odor attributes, fishy odor differed significantly among treatments (*p* < 0.05, Table S7), whereas cooked-fish, fatty, grassy, and mushroom-like odor scores did not (*p* > 0.05). The 80 °C → 100 °C treatments showed numerically higher cooked-fish and fatty odor scores than the 100 °C → 80 °C treatments. This pattern may be related to temperature-dependent formation of lipid-oxidation- and Maillard-derived volatiles, but the sensory data alone do not establish this mechanism [22].

Residual-heat treatments showed numerical differences in cooked-fish and fatty odor scores, and some residual-heat treatments had lower fishy-odor scores. These observations describe treatment-associated sensory differences but do not by themselves demonstrate a volatile-retention mechanism.

### 3.3. Quality Analysis of Perch Fish Under Single-Stage Steaming, Dual-Stage Steaming and Residual-Heat Steaming

#### 3.3.1. Changes in Shear Force

**Figure 3:**
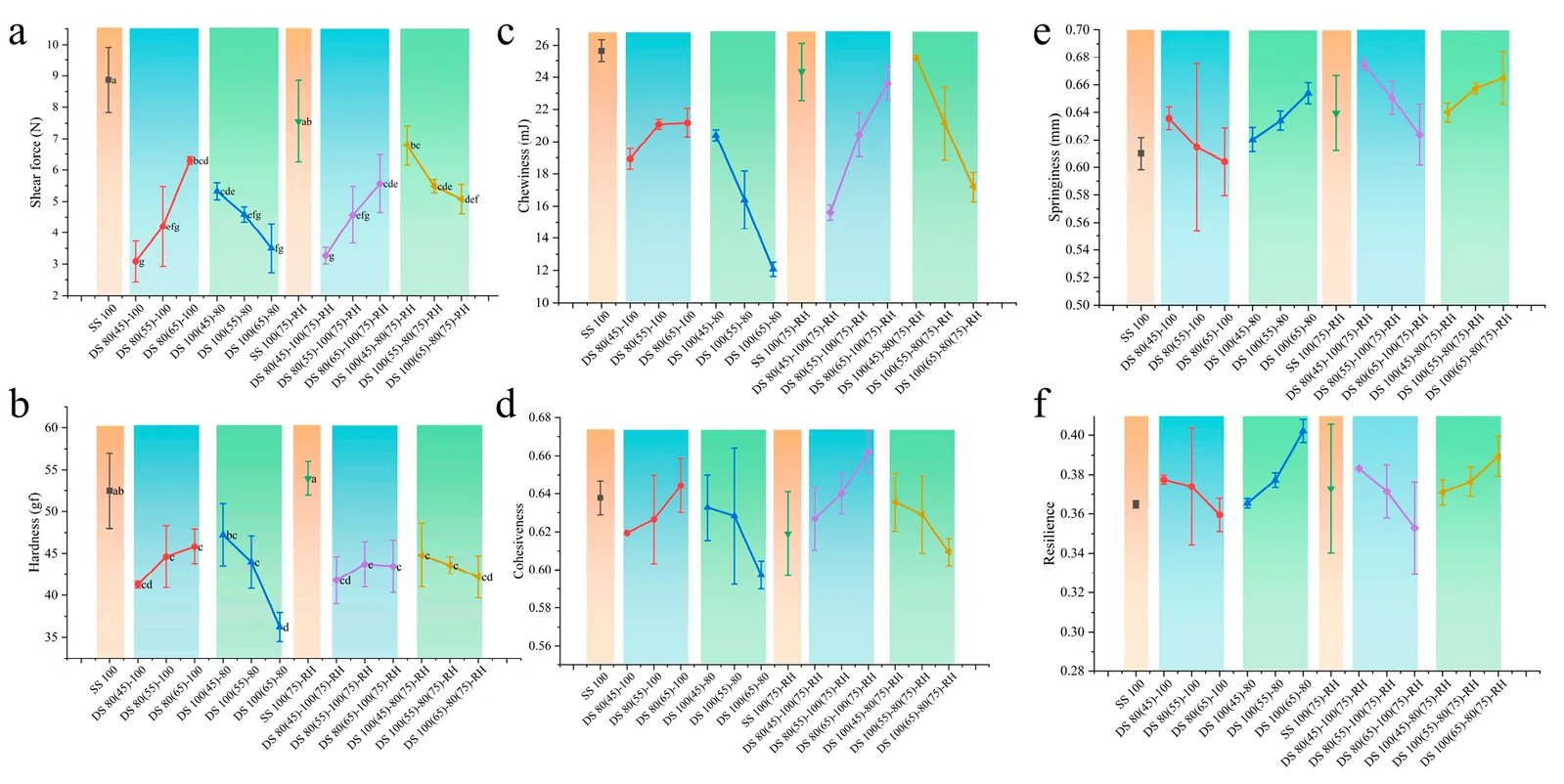
Texture properties of perch fish under different steaming modes. ((**a**): shear force; (**b**): hardness; (**c**): chewiness; (**d**): cohesiveness; (**e**): springiness; (**f**): resilience). Different lowercase letters indicate significant differences among treatments (*p* < 0.05).

Shear force is widely used to evaluate the tenderness of both raw and cooked meat, with higher values indicating a tighter and drier texture [29]. As shown in Figure 3a, the single-stage steamed sample (SS 100) exhibited the highest shear force (8.87 ± 1.04 N), whereas both dual-stage steaming and residual-heat steaming significantly reduced shear force, with values ranging from 3.09 ± 0.65 to 7.56 ± 1.30 N. This pattern is consistent with previous studies associating continuous high-temperature steaming with myofibrillar aggregation, moisture loss, and increased shear force relative to staged heating [30].

Under the 80 °C → 100 °C temperature sequence, shear force increased with higher core temperature transition points, rising from 3.09 ± 0.65 N at 45 °C to 4.20 ± 1.27 N at 55 °C, and further to 6.30 ± 0.12 N at 65 °C. This trend may be associated with myosin denaturation and muscle-fiber contraction within the 40–60 °C range [31]. A lower initial oven temperature (80 °C) required a longer time to reach the higher transition temperatures and may therefore have prolonged residence within this range. Conversely, under the 100 °C → 80 °C sequence, shear force decreased as the transition temperature increased, which may reflect a shorter residence time within the same range. These interpretations are consistent with previous thermal-processing studies of freshwater fish fillets [32,33], but protein denaturation was not measured directly in the present study. Introducing residual-heat steaming after the 80 °C → 100 °C dual-stage process had little effect at transition temperature of 45 °C (3.27 ± 0.27 N) and 55 °C (4.57 ± 0.90 N) but significantly decreased shear force at 65 °C (from 6.30 ± 0.12 N to 5.56 ± 0.91 N, *p* < 0.05), suggesting partial mitigation of texture hardening caused by prolonged high temperature exposure. In contrast, following the 100 °C → 80 °C sequence, residual-heat steaming increased shear force across all transition temperatures. For example, at transition temperatures of 45, 55, and 65 °C, shear force rose from 5.32 ± 0.27 N to 6.78 ± 0.63 N, from 4.59 ± 0.25 N to 5.48 ± 0.21 N, and from 3.51 ± 0.78 N to 5.08 ± 0.46 N, respectively. This increase is mainly related to the extended steaming duration. When the second-stage oven temperature is relatively low (80 °C), residual-heat steaming begins after the core temperature reaches 75 °C, at which point the oven temperature gradually decreases. The reduced temperature gradient slows heat transfer, prolongs the time required to reach 80 °C, and ultimately leads to increased moisture loss and reduced tenderness.

#### 3.3.2. Changes in Texture Profile Parameters

Compared with single-stage steaming, both dual-stage steaming and residual-heat steaming reduced the hardness (*p* < 0.05), cohesiveness, and chewiness of perch fish, while increasing springiness and resilience (Figure 3b–f, *p* > 0.05). In fish muscle, lower hardness, chewiness, and cohesiveness combined with higher springiness and resilience reflect a tender, smooth, and structurally cohesive texture. At a transition core temperature of 45 °C, samples processed under the 80 °C → 100 °C dual-stage steaming mode exhibited superior texture parameters than those under 100 °C → 80 °C mode, including lower hardness (41.28 ± 0.42 gf), lower chewiness (18.92 ± 0.64 mJ), lower cohesiveness (0.62 ± 0.00), and higher springiness (0.64 ± 0.01 mm) and resilience (0.38 ± 0.00). This may be attributed to the lower initial oven temperature (80 °C), which provides a more moderate heating rate, allowing myofibrillar proteins to unfold and cross-link more uniformly and form a network structure with improved water-holding capacity. In contrast, a higher initial oven temperature (100 °C) may induce rapid surface protein coagulation, hindering internal heat and moisture transfer. When the transition core temperature was increased to 55 °C, no significant differences were observed between the two temperature sequences, likely because myofibrillar protein denaturation was largely completed, reducing the impact of heating rate. At 65 °C, the 100 °C → 80 °C sequence showed lower hardness (36.22 ± 1.74 gf) and chewiness (12.07 ± 0.44 mJ), as well as higher springiness (0.65 ± 0.01 mm) and resilience (0.40 ± 0.01) than the 80 °C → 100 °C sequence (*p* < 0.05). This indicates that high heat-transfer efficiency during the initial stage helps preserve a more elastic and tender structure.

Compared with dual-stage steaming, residual-heat steaming did not significantly improve the textural properties of perch fish. This finding is consistent with a previous report [34]. The extended processing time associated with the residual-heat stage tended to reduce springiness and chewiness. Taken together, dual-stage steaming produced more favorable instrumental texture than conventional single-stage steaming in the present experiment. The observed differences may be related to the degree and rate of myofibrillar protein denaturation across the time–temperature profiles. Previous thermal-processing studies of perch and other freshwater fish fillets have similarly associated moderate multi-step heating with reduced deterioration of muscle texture relative to constant high-temperature treatment. The present results indicate that oven-temperature sequence and the core-temperature switching point are relevant to tenderness and springiness. The residual-heat stage provided limited additional textural benefit under the tested conditions.

### 3.4. Determination of Umami Compound Contents in Perch Fish Under Single-Stage Steaming, Dual-Stage Steaming and Residual-Heat Steaming

#### 3.4.1. Glutamic Acid

**Figure 4:**
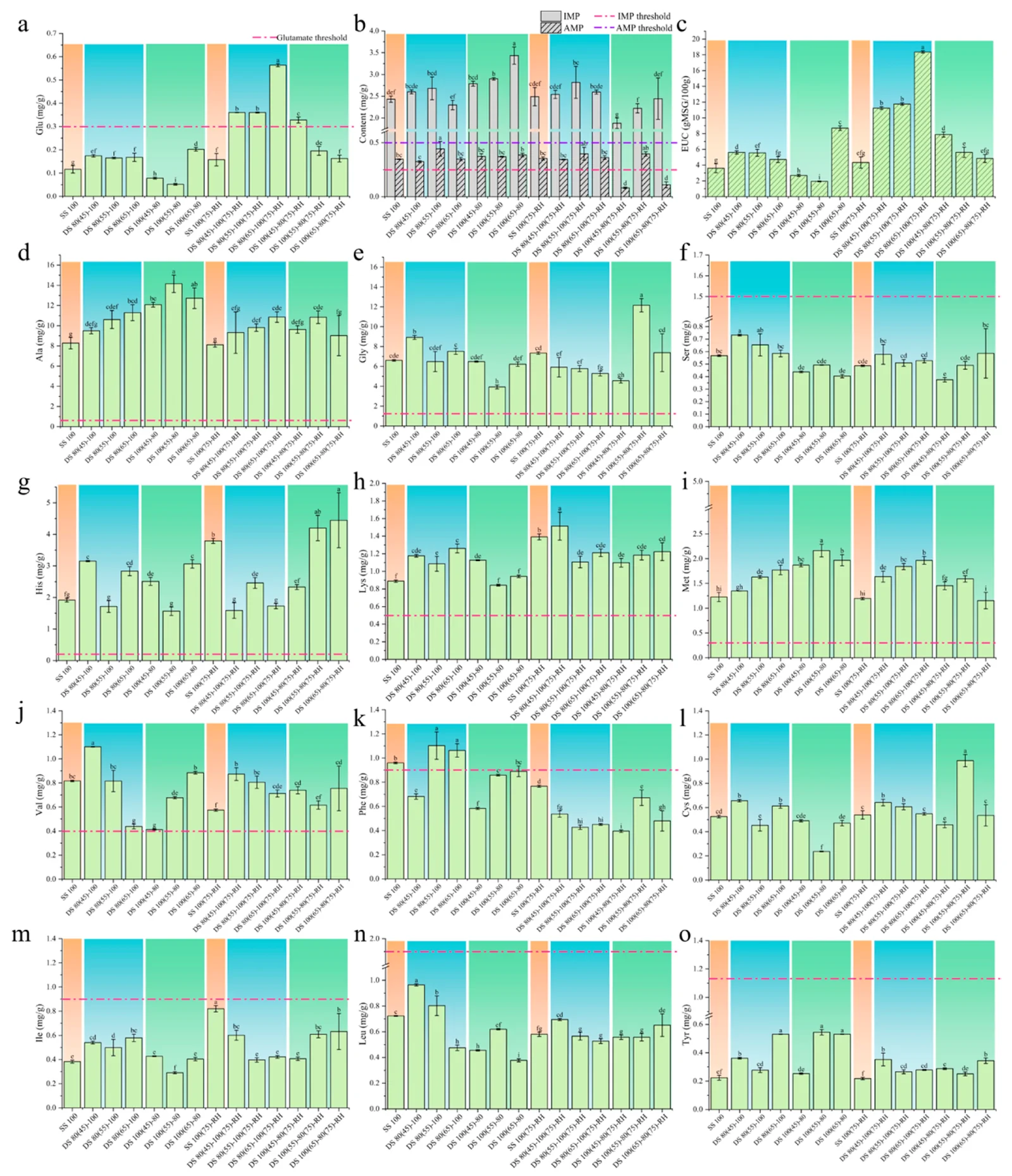
Umami-related compounds and amino acid profiles of perch fish under different steaming modes. Glutamic acid content (**a**), nucleotide content (**b**), EUC value (**c**), and other amino acids (**d**–**o**). Different lowercase letters indicate significant differences among treatments (*p* < 0.05).

Glutamic acid is the primary umami-active amino acid in steamed perch fish. As shown in Figure 4a, its content under single-stage steaming (SS 100) was 0.12 ± 0.02 mg/g. Except for a few treatments, both dual-stage steaming and residual-heat steaming increased the glutamic acid content to varying extents, ranging from 0.05 to 0.56 mg/g. Under the 80 °C → 100 °C dual-stage steaming mode, no significant differences in glutamic acid content were observed among the three transition temperatures, with values of 0.17 ± 0.01 mg/g for DS 80(45)-100, 0.16 ± 0.00 mg/g for DS 80(55)-100, and 0.17 ± 0.02 mg/g for DS 80(65)-100. In contrast, under the 100 °C → 80 °C mode, glutamic acid content was significantly influenced by the transition core temperature (*p* < 0.05). Specifically, DS 100(65)-80 exhibited a significantly higher level (0.20 ± 0.01 mg/g) than DS 100(45)-80 (0.08 ± 0.00 mg/g) and DS 100(55)-80 (0.05 ± 0.00 mg/g). Except at 65 °C, the 80 °C → 100 °C mode generally resulted in slightly higher glutamic acid contents than the 100 °C → 80 °C mode. These differences may be related to temperature-dependent protein hydrolysis and amino acid release. Previous thermal-processing research indicates that heating rate can influence endogenous protease activity and free amino acid formation [35], although protease activity was not measured in the present study.

Residual-heat steaming exerted a pronounced effect on glutamic acid accumulation. Notably, DS 80(65)-100(75)-RH exhibited the highest glutamic acid content (0.56 ± 0.01 mg/g). The gentle heating conditions during the residual-heat stage likely facilitated continued enzymatic hydrolysis and enhanced retention of glutamic acid. Given the taste threshold of glutamic acid (0.3 mg/g), only DS 80(45)-100(75)-RH, DS 80(55)-100(75)-RH, DS 80(65)-100(75)-RH, and DS 100(45)-80(75)-RH exceeded this level and could contribute directly to perceptible umami taste. Under all other conditions, glutamic acid mainly acted synergistically with flavor nucleotides such as IMP to enhance overall umami perception [12].

#### 3.4.2. Flavor Nucleotides

Two nucleotides, 5′-AMP and 5′-IMP, were detected in steamed perch fish (Figure 4b). Under all steaming conditions, IMP content was markedly higher than AMP content, indicating that IMP is the dominant umami nucleotide in perch fish. The IMP content in the SS 100 was 2.43 ± 0.07 mg/g. Dual-stage steaming slightly increased IMP content, while residual-heat steaming increased IMP content only under the 80 → 100(75) conditions. Within the dual-stage steaming modes, the 100 °C → 80 °C sequence with a transition core temperature of 65 °C yielded the highest IMP content (3.43 ± 0.20 mg/g), which was significantly higher than that of the corresponding 80 °C → 100 °C sequence. No significant differences were observed at 45 °C and 55 °C. These treatment differences may be related to temperature-dependent AMP deaminase activity and IMP degradation, although enzyme activity was not measured directly. Previous studies indicate that thermal profiles can influence endogenous enzyme activity, protein hydrolysis, and IMP formation [36]. Residual-heat steaming had no significant effect on IMP content under the tested conditions. Importantly, IMP content in all samples exceeded its taste threshold (0.25 mg/g), confirming that IMP is a major contributor to umami perception in steamed perch fish. In addition, IMP can synergistically enhance umami perception of perch fish in combination with sweet-tasting free amino acids such as alanine, glycine, and serine [37,38].

The AMP content in SS 100 was 0.35 ± 0.00 mg/g and significantly decreased under the DS 100(45)-80(75)-RH and DS 100(65)-80(75)-RH conditions, while no significant differences were observed under most other treatments. Under dual-stage steaming, DS 80(55)-100 exhibited the highest AMP content (0.44 ± 0.07 mg/g), which may be attributed to insufficient activation of AMP deaminase at the relatively low oven temperature (80 °C). Nevertheless, AMP content under all treatments remained below its taste threshold (0.5 mg/g), indicating a limited direct contribution to umami perception. Overall, dual-stage steaming, particularly DS 100(65)-80, enhanced the IMP content of steamed perch fish, whereas the residual-heat stage did not significantly promote IMP accumulation.

#### 3.4.3. Equivalent Umami Concentration Value (EUC)

EUC is used to comprehensively evaluate umami intensity by integrating the synergistic effects of umami-active amino acids and flavor nucleotides [39,40]. As shown in Figure 4c, dual-stage steaming significantly increased the EUC of perch fish compared with single-stage steaming, with DS 100(65)-80 exhibiting a relatively high value. Residual-heat steaming further elevated EUC, with DS 80(65)-100(75)-RH showing the highest value among all treatments. This enhancement is primarily attributed to the 80 °C → 100 °C temperature sequence, which favors the formation of umami compounds, while the residual-heat retention reduces their thermal loss.

### 3.5. Changes in the Contents of Other Amino Acids in Perch Fish Under Single-Stage Steaming, Dual-Stage Steaming and Residual-Heat Steaming

In addition to glutamic acid, twelve free amino acids were detected in steamed perch fish (Figure 4d–o), including three sweet-tasting amino acids (alanine, glycine, and serine) and nine bitter-tasting amino acids (histidine, lysine, methionine, valine, phenylalanine, cysteine, isoleucine, leucine, and tyrosine). Their contents were significantly influenced by both the steaming mode and the transition core temperature.

#### 3.5.1. Sweet-Tasting Amino Acids

As shown in Figure 4d–f, among sweet-tasting amino acids, alanine had the highest content, followed by glycine. The content of both alanine and glycine exceeded their taste thresholds and could directly contribute to sweetness, whereas serine remained below its taste threshold.

Dual-stage steaming was associated with treatment-dependent differences in alanine content, consistent with previous studies [41]. Under the 80 °C → 100 °C mode, alanine content increased with increasing transition temperature. This pattern may reflect differences in protein hydrolysis and amino acid degradation across the time–temperature profiles, although enzyme activity was not measured directly. Under the 100 °C → 80 °C mode, alanine content initially increased and then decreased as the transition core temperature rose. Differences in heating duration and thermal exposure may have contributed to this pattern.

#### 3.5.2. Bitter-Tasting Amino Acids

The contents of histidine and lysine under dual-stage steaming showed a decreasing-increasing trend with increasing transition core temperature (Figure 4g,h). At 45–55 °C, elevated temperature accelerated the Maillard reaction, coupled with thermal degradation and protein denaturation, which led to a significant decrease in amino acid contents. Notably, this reduction was more pronounced in the 100 °C → 80 °C group. In the range of 55–65 °C, extensive protein denaturation and hydrolysis became dominant, increasing amino acid release. Compared with the 80 °C → 100 °C group, the 100 °C → 80 °C group exhibited a pronounced rebound, benefiting from the combined effects of initial high-temperature denaturation and subsequent moderate-temperature treatment, which facilitated deeper protein hydrolysis. Methionine exhibited distinct responses (Figure 4i). At the same transition point, its content was significantly higher under the 100 °C → 80 °C mode than that under the 80 °C → 100 °C mode. In the 80 °C → 100 °C mode, its content increased monotonically with increasing transition core temperatures, whereas under the 100 °C → 80 °C mode it increased at lower transition temperatures but decreased at higher ones due to protease inactivation and intensified thermal degradation. Additionally, the introduction of residual-heat steaming generally resulted in lower methionine content.

Among the bitter-tasting amino acids, histidine, methionine, lysine, and valine exceeded their respective taste thresholds and may interact synergistically with umami and sweet amino acids, contributing to the characteristic and complex taste profile of fish meat [42,43]. Overall, residual-heat retention had limited effects on the contents of most amino acids, likely because of its short duration and reduced heat transfer, while extended heating may have slightly decreased free amino acid contents through moisture loss.

### 3.6. Changes in the Composition and Content of Aroma Compounds Under Single-Stage Steaming, Dual-Stage Steaming and Residual-Heat Steaming

**Figure 5:**
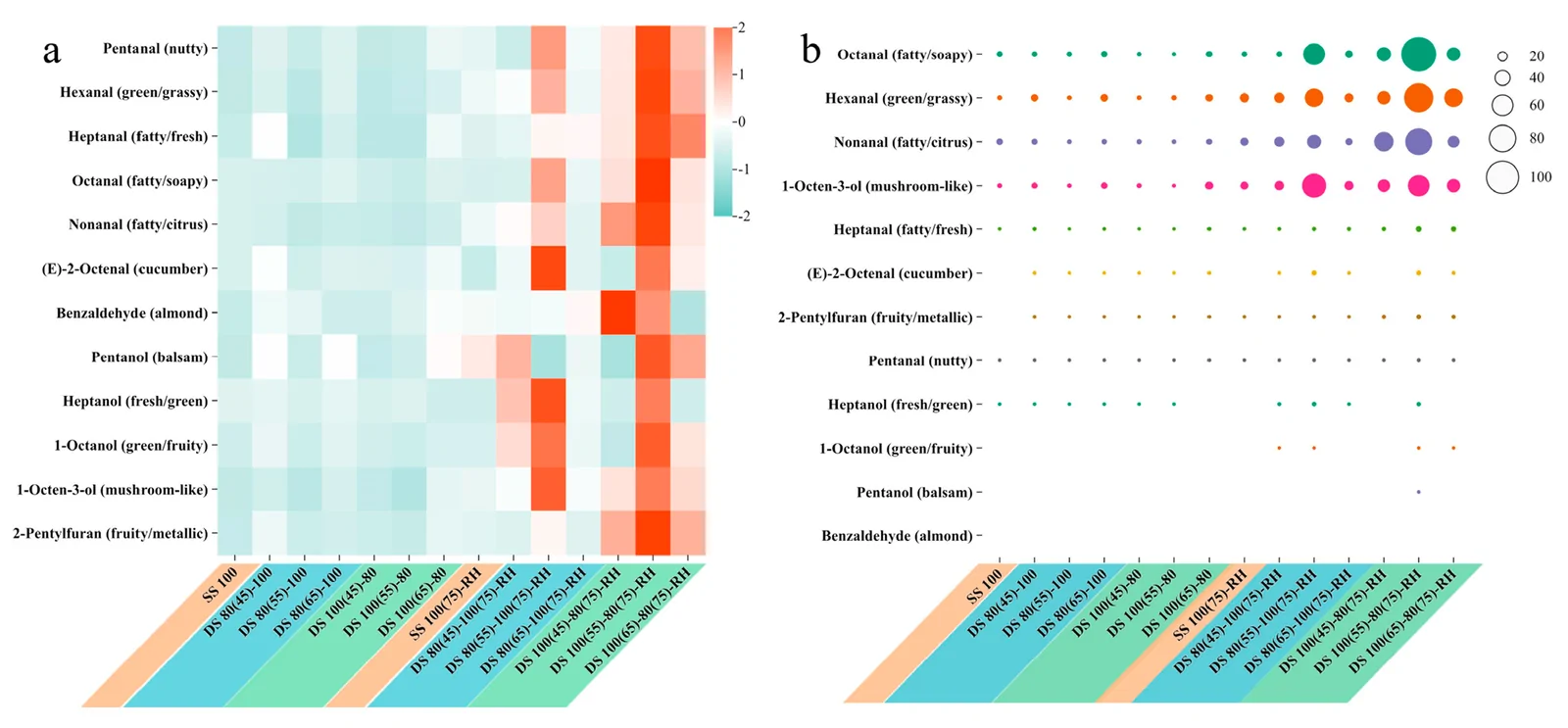
Heatmap of aroma compound content (**a**) and the rOAV (**b**) of perch fish under different steaming modes.

Aroma compounds play a crucial role in determining the characteristic flavor of steamed perch fish. As shown in Figure 5a, a total of 12 volatile aroma compounds were identified under different steaming conditions, including 7 aldehydes, 4 alcohols, and 1 furan. Compared with single-stage steaming, both dual-stage and residual-heat steaming significantly increased the total aroma content, with residual-heat steaming demonstrating the most pronounced enhancement (*p* < 0.05). Under the residual-heat steaming mode, the 100 °C → 80 °C sequence produced higher total volatile contents than the 80 °C → 100 °C sequence, reaching its peak when the transition core temperature was set at 55 °C. This pattern is consistent with the temperature-dependent formation of aroma compounds reported by Chang et al. [44]. It may reflect differences in the formation and conversion of lipid- and amino-acid-derived precursors across the time–temperature profiles [45]; these mechanisms were not measured directly in the present study. Compared with dual-stage steaming, residual-heat steaming terminates active oven heating at a core temperature of 75 °C and relies on heat stored in the closed chamber for further cooking. The lower temperature, reduced steam flow, and enclosed chamber could affect volatilization and condensation; however, these processes were not measured directly. Accordingly, the higher measured volatile concentrations are interpreted as treatment associations rather than direct evidence of enhanced retention.

The contribution of individual compounds to overall aroma was evaluated using relative odor activity values (rOAVs) (Figure 5b, Table S2). Octanal (fatty) exhibited the highest rOAV, followed by hexanal (grassy), nonanal (fatty), and 1-octen-3-ol (mushroom-like), indicating that these compounds are the key aroma contributors in steamed perch fish. Aldehydes were the dominant aroma compounds in both diversity and abundance. The highest total aldehyde content (676.17 ± 75.10 ng/g) was observed under DS 100(55)-80(75)-RH, whereas DS 100(45)-80 showed the lowest level (37.98 ± 11.07 ng/g). Aldehydes such as hexanal, octanal, and nonanal are commonly associated with lipid oxidation of polyunsaturated fatty acids [46,47,48]. The observed treatment differences may reflect temperature-dependent precursor formation and oxidation, although these pathways were not measured directly. Among alcohols, 1-octen-3-ol, a product associated with the oxidation of linoleic and arachidonic acid [49,50], showed the highest content under DS 80(55)-100(75)-RH (72.20 ± 11.52 ng/g), followed by DS 100(55)-80(75)-RH (63.24 ± 3.72 ng/g). These measured differences do not directly demonstrate enhanced retention. 2-Pentylfuran, which is associated with linoleic acid oxidation and fruity and metallic notes [51], was highest under DS 100(55)-80(75)-RH (25.15 ± 0.28 ng/g), followed by DS 100(45)-80(75)-RH (13.90 ± 1.89 ng/g).

### 3.7. Multivariate Analysis of Quality Compounds in Perch Fish Under Single-Stage Steaming, Dual-Stage Steaming and Residual-Heat Steaming

#### 3.7.1. Correlation Analysis of Quality Indicators

**Figure 6:**
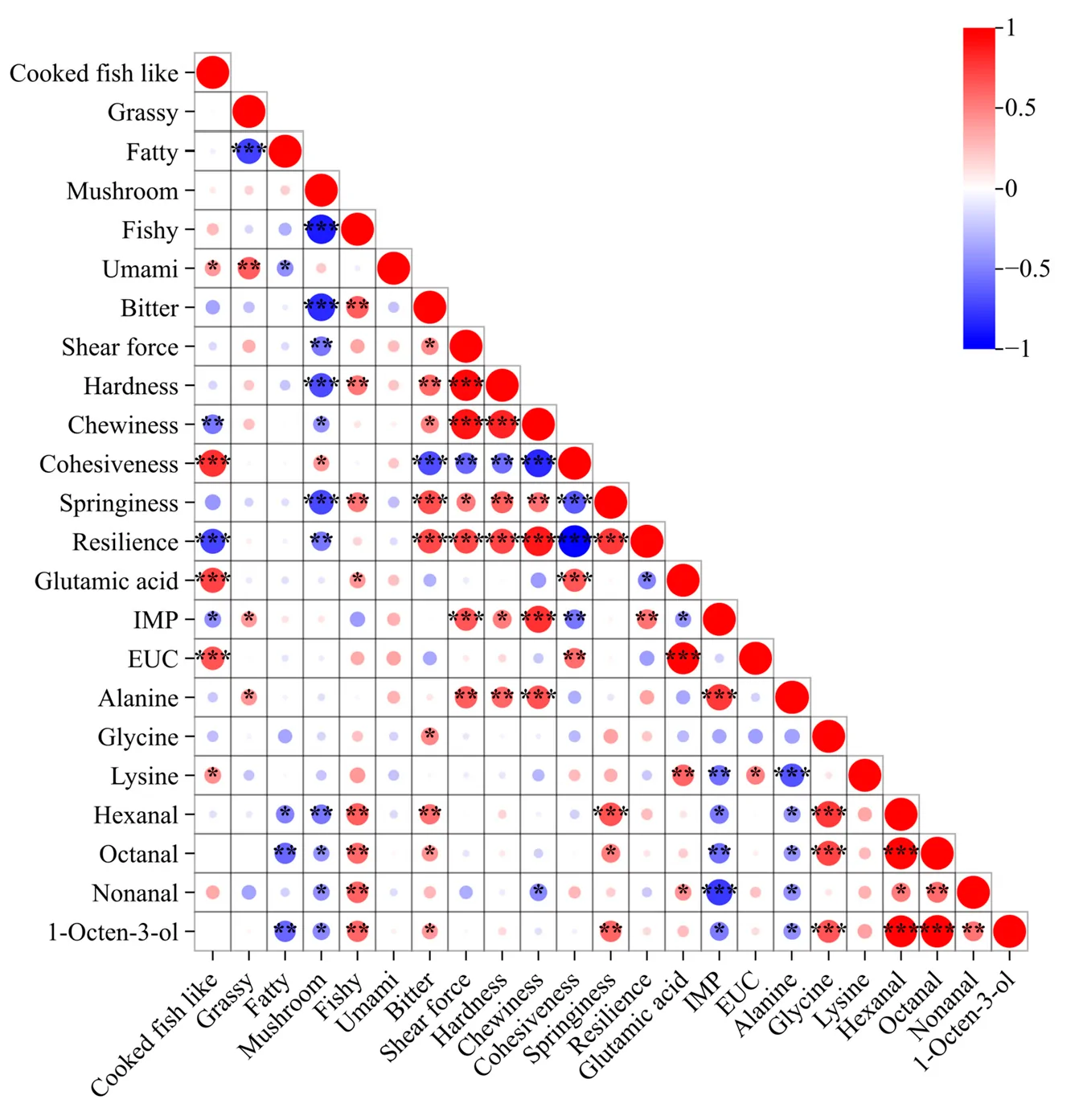
Heatmap of correlations among quality indicators of perch fish under different steaming modes. Pearson’s correlation coefficient was used for the analysis. Asterisks indicate significant correlations: * *p* < 0.05, ** *p* < 0.01, and *** *p* < 0.001.

The correlation analysis of quality indicators under different steaming conditions is presented in Figure 6. In terms of sensory evaluation, grassy odor showed a significant negative correlation with fatty odor, while mushroom-like odor showed a highly significant negative correlation with fishy odor and bitterness. These associations indicate that pleasant and undesirable odor notes varied inversely across the tested treatments [52]. For texture-related parameters, shear force exhibited a highly significant positive correlation with hardness and chewiness, indicating that higher mechanical resistance was associated with a tougher texture. Cohesiveness demonstrated a significant negative correlation with chewiness and a highly significant negative correlation with resilience. Such inter-relationships among TPA parameters are commonly observed in thermally treated fish muscle, as protein denaturation jointly drives changes in multiple texture metrics [53]. Regarding taste-related indices, EUC showed a highly positive correlation with glutamic acid content [54].

Overall, the strong intercorrelations among multiple quality indicators indicated overlapping information among variables. Therefore, principal component analysis (PCA) was used for dimensionality reduction.

#### 3.7.2. Principal Component Analysis and Comprehensive Evaluation Model

A total of 27 quality indicators were determined in this study. Based on the correlation matrix, eight representative indicators (the fatty sensory score, shear force, springiness, IMP, alanine, hexanal, octanal, and 1-octen-3-ol) were retained for PCA to reduce redundant information while maintaining coverage of sensory perception, texture, umami-related compounds, free amino acids, and key volatile compounds. All variables were subjected to Z-score standardization prior to PCA. Three principal components with eigenvalues greater than 1 were extracted, accounting for 86.1% of total variance. The first principal component (PC1), which explained nearly 40% of the total variance, was primarily associated with key aroma compounds and umami-related indicators, reflecting the overall flavor characteristics of steamed perch fish. PC2 and PC3 mainly represented textural properties and specific taste attributes, respectively.

Based on the PCA results, principal component scores were calculated as follows:

(3) F_1_ = −0.4279·X_1_ − 0.0083·X_2_ − 0.097·X_3_ − 0.0933·X_4_ + 0.1591·X_5_ + 0.2915·X_6_ + 0.2272·X_7_ + 0.2169·X_8_

(4) F_2_ = −0.1921·X_1_ + 0.3784·X_2_ + 0.0575·X_3_ + 0.3647·X_4_ + 0.4064·X_5_ − 0.0326·X_6_ − 0.0338·X_7_ − 0.0539·X_8_

(5) F_3_ = 0.4293·X_1_ + 0.2455·X_2_ + 0.6096·X_3_ + 0.1301·X_4_ − 0.3055·X_5_ − 0.0114·X_6_ + 0.1230·X_7_ + 0.1389·X_8_

where X_1_–X_8_ represent fatty taste, shear force, springiness, IMP, alanine, hexanal, octanal and 1-octen-3-ol, respectively.

A comprehensive quality evaluation model for perch fish quality was then established by weighting each principal component according to its variance contribution:

(6) F = 0.4648·F_1_ + 0.2580·F_2_ + 0.1382·F_3_

where F_1_–F_3_ denote the scores of the three principal components; and F is the comprehensive quality score.

Based on this model, standardized data were substituted to calculate F_1_, F_2_, F_3_, and the comprehensive score F. The ranking results are presented in Table 2. The top three conditions were within the residual-heat group, and DS 100(55)-80(75)-RH had the highest descriptive comprehensive score (0.52). This PCA ranking summarizes the measured variables but does not constitute an inferential test of significant superiority. The result identifies DS 100(55)-80(75)-RH as a promising condition within the tested laboratory-scale profiles; its performance and feasibility require validation under larger-scale production conditions.

## 4. Conclusions

This study systematically compared single-stage, dual-stage and residual-heat steaming strategies to elucidate their effects on the texture, taste and aroma characteristics of perch fish based on comprehensive analytical data.

Compared with single-stage steaming, dual-stage treatments significantly reduced shear force and hardness and produced treatment-dependent differences in umami-related compounds (*p* < 0.05 for variables showing treatment effects). Several volatile aroma compounds showed significant treatment effects (*p* < 0.05) and had higher measured concentrations under residual-heat steaming. PCA assigned the highest descriptive comprehensive score (0.52) to DS 100(55)-80(75)-RH; this ranking was not an inferential significance test. These findings identify promising time–temperature profiles for balancing texture, taste, and aroma in steamed perch fish under the tested laboratory conditions.

Nevertheless, each treatment was evaluated in three independent laboratory-scale heating batches, and storage, equipment scale-up, and batch catering conditions were not examined. Further studies should use larger independent batches, directly measure the proposed mechanisms, and evaluate reproducibility, energy use, safety, and sensory performance under practical production conditions.

## Figures and Tables

**Table 1:** Steaming modes and processing parameters for perch fish samples.

Steaming Mode	Oven Panel Temperature (°C)			Core Temperature at the Switching Point (°C)		Heating Status of the Oven	Core Temperature at the Steaming Endpoint (°C)	Abbreviation Rule
	First Stage	Second Stage	Third Stage					
Single-stage Steaming (SS)	100	100	100	-	-	Continuous Heating	80	SS 100
Dual-stage Steaming (DS)	80	100	100	45	-	Continuous Heating	80	DS 80(45)-100
				55	-			DS 80(55)-100
				65	-			DS 80(65)-100
	100	80	80	45	-	Continuous Heating	80	DS 100(45)-80
				55	-			DS 100(55)-80
				65	-			DS 100(65)-80
Single-stage Steaming + Residual-heat steaming (RH)	100	100	Residual Heat	-	75	Stop heating when the core temperature reaches 75 °C	80	SS 100(75)-RH
Dual-stage Steaming + Residual-heat steaming (RH)	80	100	Residual Heat	45	75	Stop heating when the core temperature reaches 75 °C	80	DS 80(45)-100(75)-RH
				55	75			DS 80(55)-100(75)-RH
				65	75			DS 80(65)-100(75)-RH
	100	80	Residual Heat	45	75	Stop heating when the core temperature reaches 75 °C	80	DS 100(45)-80(75)-RH
				55	75			DS 100(55)-80(75)-RH
				65	75			DS 100(65)-80(75)-RH

**Table 2:** Factor scores and ranking of perch fish quality indicators under different steaming modes.

	F1	F2	F3	F	Comprehensive Ranking
DS 100(55)-80(75)-RH	0.68	0.41	0.73	0.52	1
DS 80(55)-100(75)-RH	0.49	0.57	0.78	0.48	2
DS 100(65)-80	−0.12	0.99	0.73	0.30	3
DS 100(65)-80(75)-RH	−0.02	0.30	1.12	0.22	4
DS 80(55)-100	0.02	0.67	0.23	0.21	5
DS 80(65)-100	0.14	0.47	0.01	0.19	6
DS 100(45)-80(75)-RH	0.14	0.16	0.56	0.18	7
DS 100(55)-80	−0.23	0.82	0.51	0.18	8
DS 80(45)-100(75)-RH	−0.27	0.51	1.19	0.17	9
DS 80(45)-100	−0.26	0.52	0.83	0.13	10
DS 80(65)-100(75)-RH	−0.27	0.46	0.52	0.06	11
DS 100(45)-80	−0.39	0.53	0.60	0.04	12
SS 100(75)-RH	−0.28	0.11	0.74	0.00	13
SS 100	−0.43	−0.05	0.52	−0.14	14

## Data Availability

The original contributions presented in the study are included in the article/Supplementary Materials. Further inquiries can be directed to the corresponding author.

## Supplementary Materials

The following supporting information can be downloaded at https://www.mdpi.com/article/10.3390/foods15183264/s1. Figure S1: Representative photographs of raw perch fish and perch fish treated with different steaming modes; Tables S1–S8: Detailed experimental parameters and statistical analysis data. Reference [55] is cited in the supplementary materials.

## Abbreviations

The following abbreviations are used in this manuscript:

HPLC	High-performance liquid chromatography
GC-O-MS	Gas chromatography–olfactometry–mass spectrometry
5′-AMP	Adenosine 5′-monophosphate disodium salt
5′-IMP	Inosine 5′-monophosphate disodium salt
5′-GMP	Guanosine 5′-monophosphate disodium salt
OPA	o-Phthalaldehyde
FMOC	9-fluorenylmethyl chloroformate
TPA	Texture profile analysis
EUC	Equivalent umami concentration
rOAV	Relative odor activity value analysis
