# Supplementary material for: Effect of Time–Temperature Modulation Combined with Residual Heating on Flavor and Texture Attributes of Steamed Perch Fish
Source: Foods. 2026 Sep 16;15(18):3264. doi: 10.3390/foods15183264 (PMC13605891; doi:10.3390/foods15183264)
Supplement: Supplementary file 1 [file foods-15-03264-s001.zip › foods-4534961-supplementary.pdf]

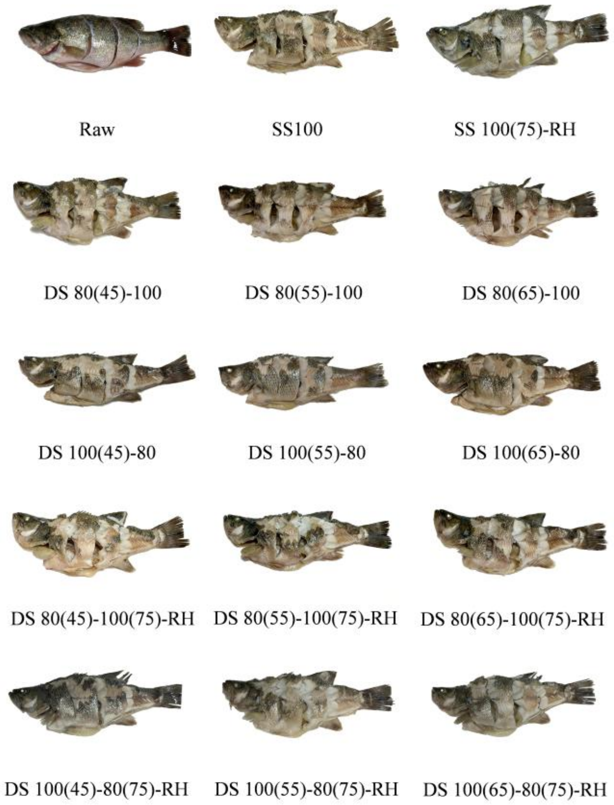


**Figure S1.** Representative photographs of raw perch fish and perch fish treated with different steaming modes. Specific sample codes correspond to those defined in Table 1.

**Table S1** Scoring criteria for appearance and sensory evaluation of perch fish under different steaming modes.

| | Evaluation Indicator | Standards | Scores |
|---|---|---|---|
| Appearance | Color (15 points) | Normal white color with shiny muscle cross-section | 13-15 |
| | | White color with relatively shiny muscle cross-section | 10-12 |
| | | Slightly dull color with faint luster on muscle cross-section | 7-9 |
| | | Dull color with no luster on muscle cross-section | 0-6 |
| | Structure (10 points) | Clear texture, easy separation of meat and bones, dense and intact muscle tissue | 8-10 |
| | | Local slightly loose muscle tissue, or slightly difficult separation of meat and bones | 5-7 |
| | | Excessively loose muscle tissue, or excessively tight tissue leading to difficult separation of meat and bones | 0-4 |
| | Springiness (10 points) | Firm and elastic, indentation disappears immediately after pressing with hand | 8-10 |
| | | Slightly poor elasticity, indentation disappears slowly after pressing with hand | 5-7 |
| | | Poor elasticity, indentation does not disappear after pressing with hand | 0-4 |
| | Completeness (15 points) | Intact and shiny fish skin | 10-15 |
| | | Slightly incomplete fish skin or relatively dull color | 5-9 |
| | | Severely cracked fish skin | 0-4 |
| Texture | Tenderness (25 points) | Tender meat texture | 19-25 |
| | | Slightly tough meat texture | 13-18 |
| | | Dry meat texture with tough taste | 7-12 |
| | | Dry meat texture with strong tough taste | 0-6 |
| | Juiciness (10 points) | Abundant juice release, persistent juicy sensation | 8-10 |
| | | Moderate juice release, general juicy taste | 6-7 |
| | | Rare juice release, dry and crumbly mouthfeel | 0-5 |
| | Chewiness (15 points) | Moderate chewiness when eating fish meat | 10-15 |
| | | Slightly weak or slightly strong chewiness when eating fish meat | 5-9 |
| | | No chewiness or excessively strong chewiness when eating fish meat | 0-4 |

**Table S2** Sensory evaluation scores of steamed perch fish under different steaming modes

| | Color | Completeness | Structure | Springiness | Tenderness | Chewiness | Juiciness | Cooked fish | Grassy | Fatty | Mushroom -like | Fishy | Umami | Bitter |
|---|---|---|---|---|---|---|---|---|---|---|---|---|---|---|
| SS 100 | 11±2.5 | 12±1.2 | 7±1.2 | 7±1.6 | 16±4.4 | 9±3.0 | 7±2.0 | 3±0.9 | 1±0.9 | 3±1.0 | 2±1.1 | 4±0.7a | 3±0.8 | 1±0.8 |
| DS 80(45)-100 | 11±3.3 | 9±2.5 | 8±0.4 | 7±1.6 | 19±2.4 | 10±2.3 | 8±1.2 | 4±0.7 | 2±1.0 | 2±0.5 | 1±0.8 | 2±0.8bc | 3±1.2 | 1±0.6 |
| DS 80(55)-100 | 12±1.3 | 10±1.6 | 8±0.5 | 7±1.1 | 20±3.9 | 12±0.8 | 8±1.5 | 4±0.4 | 2±1.1 | 1±0.5 | 1±0.4 | 2±1bcd | 4±0.9 | 1±1.0 |
| DS 80(65)-100 | 12±2.7 | 12±2.2 | 9±1.0 | 8±0.8 | 17±5.2 | 11±2.4 | 8±1.2 | 4±0.9 | 2±1.0 | 1±0.8 | 1±0.7 | 2±1.4bc | 3±0.7 | 1±0.8 |
| DS 100(45)-80 | 11±2.4 | 11±2.4 | 8±0.4 | 7±0.7 | 17±1.7 | 10±2.5 | 7±0.7 | 4±0.4 | 1±0.7 | 3±0.9 | 1±1.4 | 2±1.0bcd | 4±0.9 | 1±0.9 |
| DS 100(55)-80 | 12±2.5 | 11±3.1 | 9±0.5 | 8±1.2 | 18±4.3 | 11±2.7 | 7±1.3 | 3±0.7 | 2±1.0 | 2±1.0 | 1±0.6 | 2±0.8bcd | 3±0.0 | 1±0.7 |
| DS 100(65)-80 | 11±3.6 | 10±3.3 | 8±0.8 | 7±1.9 | 18±4.5 | 10±2.2 | 8±1.5 | 3±0.4 | 2±0.7 | 2±0.8 | 1±0.7 | 3±1.0abc | 4±0.4 | 1±0.5 |
| SS 100(75)-RH | 10±3.6 | 9±4.3 | 8±1.4 | 6±2.4 | 15±4.7 | 9±2.5 | 7±2.0 | 3±1.2 | 2±0.9 | 2±0.7 | 2±0.9 | 3±1.2ab | 3±0 | 1±1.1 |
| DS 80(45)-100(75)-RH | 11±3.1 | 10±2.8 | 8±1.2 | 8±1.9 | 14±3.4 | 11±1.2 | 7±1.0 | 4±0.9 | 1±0.6 | 2±0.9 | 1±0.7 | 2±1.0bcd | 3±0.4 | 1±0.5 |
| DS 80(55)-100(75)-RH | 13±2.4 | 12±2.4 | 8±0.8 | 8±0.9 | 18±4.4 | 11±1.8 | 8±1.2 | 4±0.6 | 2±1.0 | 1±0.8 | 1±0.6 | 2±1.3bcd | 4±0.4 | 0±0.4 |
| DS 80(65)-100(75)-RH | 12±1.7 | 12±1.7 | 8±1.1 | 8±0.8 | 18±2.3 | 10±2.7 | 6±1.5 | 4±0.3 | 1±1.1 | 2±0.9 | 1±0.5 | 1±1.4cd | 3±0.5 | 0±0.5 |
| DS 100(45)-80(75)-RH | 12±2.0 | 12±2.2 | 8±0.8 | 8±1.8 | 18±2.3 | 13±1.9 | 8±0.8 | 4±0.8 | 1±0.9 | 2±0.7 | 1±0.4 | 1±1.0cd | 3±1.0 | 1±0.9 |
| DS 100(55)-80(75)-RH | 10±4.1 | 10±4.5 | 8±0.5 | 7±2.5 | 18±2.6 | 11±1.7 | 8±1.3 | 4±0.7 | 1±0.0 | 1±0.5 | 1±1.5 | 1±1.0cd | 3±0.9 | 0±0.5 |
| DS 100(65)-80(75)-RH | 13±2.9 | 10±5.0 | 8±1.2 | 7±1.2 | 17±3.6 | 9±2.5 | 7±1.7 | 4±1.0 | 1±0.8 | 2±0.8 | 0±0.3 | 1±0.5d | 4±1.4 | 0±0.0 |
| F-value | 0.615 | 0.847 | 0.972 | 0.941 | 0.782 | 1.340 | 0.952 | 1.269 | 0.354 | 1.282 | 1.156 | 3.094 | 1.194 | 0.701 |
| *p*-value | 0.833 | 0.611 | 0.488 | 0.517 | 0.675 | 0.214 | 0.507 | 0.255 | 0.979 | 0.248 | 0.332 | 0.001 | 0.307 | 0.755 |

Values are expressed as mean ± standard deviation. Different superscript lowercase letters within the same column indicate significant differences ($p < 0.05$).

**Table S3** Textural parameters of steamed perch fish under different steaming modes.

| Parameters | Shear force | Hardness | Chewiness | Cohesiveness | Springiness | Resilience |
|---|---|---|---|---|---|---|
| SS 100 | 8.87±1.04a | 52.47±4.51ab | 25.65±0.70 | 0.64±0.01 | 0.61±0.01 | 0.36±0.00 |
| DS 80(45)-100 | 3.09±0.65g | 41.28±0.42cd | 18.92±0.64 | 0.62±0.00 | 0.64±0.01 | 0.38±0.00 |
| DS 80(55)-100 | 4.20±1.27efg | 44.62±3.69c | 21.09±0.31 | 0.62±0.02 | 0.61±0.06 | 0.37±0.03 |
| DS 80(65)-100 | 6.30±0.12bcd | 45.84±2.06c | 21.18±0.88 | 0.64±0.01 | 0.60±0.02 | 0.36±0.01 |
| DS 100(45)-80 | 5.32±0.27cde | 47.22±3.71bc | 20.40±0.34 | 0.63±0.01 | 0.62±0.01 | 0.37±0.00 |
| DS 100(55)-80 | 4.59±0.25efg | 43.97±3.14c | 16.38±1.79 | 0.63±0.04 | 0.63±0.01 | 0.38±0.00 |
| DS 100(65)-80 | 3.51±0.78fg | 36.22±1.74d | 12.07±0.44 | 0.60±0.01 | 0.65±0.01 | 0.40±0.01 |
| SS 100(75)-RH | 7.56±1.30ab | 53.97±2.04a | 24.33±1.79 | 0.62±0.02 | 0.64±0.03 | 0.37±0.03 |
| DS 80(45)-100(75)-RH | 3.27±0.27g | 41.79±2.81cd | 15.62±0.48 | 0.63±0.02 | 0.67±0.00 | 0.38±0.00 |
| DS 80(55)-100(75)-RH | 4.57±0.90efg | 43.70±2.70c | 20.43±1.37 | 0.64±0.01 | 0.65±0.01 | 0.37±0.01 |
| DS 80(65)-100(75)-RH | 5.56±0.91cde | 43.46±3.13c | 23.61±1.03 | 0.66±0.01 | 0.62±0.02 | 0.35±0.02 |
| DS 100(45)-80(75)-RH | 6.78±0.63bc | 44.80±3.78c | 25.20±0.19 | 0.64±0.02 | 0.64±0.01 | 0.37±0.01 |
| DS 100(55)-80(75)-RH | 5.48±0.21cde | 43.59±0.98c | 21.12±2.27 | 0.63±0.02 | 0.66±0.00 | 0.38±0.01 |
| DS 100(65)-80(75)-RH | 5.08±0.46def | 42.19±2.50cd | 17.18±0.91 | 0.61±0.01 | 0.67±0.02 | 0.39±0.01 |
| F-value | 11.387 | 5.349 | 2.283 | 1.737 | 2.061 | 1.525 |
| *p*-value | <0.001 | 0.001 | 0.064 | 0.153 | 0.091 | 0.216 |

Values are expressed as mean ± standard deviation. Different superscript lowercase letters within the same column indicate significant differences ($p < 0.05$).

**Table S4.** Contents of umami-related compounds and equivalent umami concentration (EUC) of steamed perch fish under different steaming modes.

| | Glu (mg/g) | IMP (mg/g) | AMP (mg/g) | EUC (gMSG/100g) |
|---|---|---|---|---|
| SS 100 | 0.12±0.02g | 2.43±0.07def | 0.35±0.00bc | 3.60±0.56g |
| DS 80(45)-100 | 0.17±0.01ef | 2.60±0.05bcde | 0.32±0.01c | 5.61±0.21d |
| DS 80(55)-100 | 0.16±0.00f | 2.68±0.26bcd | 0.44±0.07a | 5.56±0.44ef |
| DS 80(65)-100 | 0.17±0.02f | 2.30±0.11ef | 0.35±0.02bc | 4.71±0.38fg |
| DS 100(45)-80 | 0.08±0.00h | 2.79±0.06bcd | 0.37±0.02bc | 2.69±0.13h |
| DS 100(55)-80 | 0.05±0.00i | 2.90±0.03b | 0.37±0bc | 1.93±0.05i |
| DS 100(65)-80 | 0.2±0.01d | 3.43±0.20a | 0.39±0.02b | 8.70±0.30c |
| SS 100(75)-RH | 0.16±0.03f | 2.49±0.21cdef | 0.35±0.01bc | 4.33±0.71efg |
| DS 80(45)-100(75)-RH | 0.36±0.00b | 2.54±0.09cdef | 0.34±0.00bc | 11.23±0.17b |
| DS 80(55)-100(75)-RH | 0.36±0.00b | 2.82±0.37bc | 0.4±0.06ab | 11.75±0.15b |
| DS 80(65)-100(75)-RH | 0.56±0.01a | 2.6±0.05bcde | 0.36±0.02bc | 18.36±0.14a |
| DS 100(45)-80(75)-RH | 0.33±0.01c | 1.88±0.12g | 0.08±0.01d | 7.88±0.32d |
| DS 100(55)-80(75)-RH | 0.20±0.02de | 2.22±0.11f | 0.4±0.02ab | 5.62±0.65e |
| DS 100(65)-80(75)-RH | 0.16±0.01f | 2.44±0.48def | 0.11±0.03d | 4.84±0.51efg |
| F-value | 317.981 | 5.318 | 39.600 | 248.916 |
| *p*-value | <0.001 | <0.001 | <0.001 | <0.001 |

Values are expressed as mean ± standard deviation. Different superscript lowercase letters within the same column indicate significant differences ($p < 0.05$).

**Table S5** Free amino acid contents of steamed perch fish under different steaming treatments.

| | Content (mg/g) | | | | | | | | | | | |
|---|---|---|---|---|---|---|---|---|---|---|---|---|
| | Ala | Gly | Ser | His | Lys | Met | Val | Phe | Cys | Ile | Leu | Tyr |
| SS 100 | 8.27±0.56g | 6.61±0.08cde | 0.57±0.01bc | 1.92±0.06fg | 0.89±0.01f | 1.22±0.09hi | 0.82±0.01bc | 0.96±0.01b | 0.53±0.01cd | 0.38±0.01e | 0.72±0.00c | 0.22±0.02ef |
| DS 80(45)-100 | 9.50±0.31defg | 8.92±0.2b | 0.73±0.01a | 3.15±0.02c | 1.18±0.02cde | 1.35±0.00gh | 1.10±0.00a | 0.68±0.02e | 0.66±0.01b | 0.54±0.01cd | 0.96±0.01a | 0.36±0.01b |
| DS 80(55)-100 | 10.61±0.9cdef | 6.48±1.01cdef | 0.65±0.09ab | 1.71±0.19g | 1.09±0.08e | 1.63±0.03de | 0.82±0.09bc | 1.10±0.11a | 0.45±0.05e | 0.50±0.07d | 0.80±0.08b | 0.28±0.02cd |
| DS 80(65)-100 | 11.29±0.79bcd | 7.52±0.3c | 0.59±0.03bc | 2.83±0.14cd | 1.26±0.05c | 1.77±0.10cd | 0.44±0.02g | 1.06±0.05a | 0.61±0.02b | 0.58±0.03bc | 0.47±0.02h | 0.53±0.00a |
| DS 100(45)-80 | 12.08±0.24bc | 6.49±0.07cdef | 0.44±0.01de | 2.51±0.13de | 1.13±0.01de | 1.87±0.04bc | 0.41±0.01g | 0.58±0.01f | 0.49±0.01cde | 0.43±0.00e | 0.46±0.00h | 0.25±0.01de |
| DS 100(55)-80 | 14.15±0.86a | 3.93±0.19h | 0.49±0.00cde | 1.56±0.14g | 0.84±0.01f | 2.16±0.13a | 0.68±0.01de | 0.86±0.01c | 0.24±0.00f | 0.29±0.01f | 0.62±0.00ef | 0.54±0.02a |
| DS 100(65)-80 | 12.72±1.02ab | 6.23±0.23def | 0.40±0.01de | 3.06±0.14c | 0.94±0.01f | 1.97±0.11b | 0.89±0.01b | 0.89±0.04bc | 0.47±0.02de | 0.40±0.01e | 0.38±0.01i | 0.53±0.00a |
| SS 100(75)-RH | 8.11±0.23g | 7.34±0.12cd | 0.49±0.01cde | 3.79±0.08b | 1.39±0.03b | 1.20±0.02hi | 0.57±0.01f | 0.77±0.01d | 0.54±0.03c | 0.82±0.03a | 0.58±0.02fg | 0.22±0.01f |
| DS 80(45)-100(75)-RH | 9.32±2.06efg | 5.92±0.98ef | 0.58±0.08bc | 1.59±0.25g | 1.51±0.16a | 1.64±0.11de | 0.87±0.05b | 0.54±0.03fg | 0.64±0.03b | 0.60±0.04bc | 0.69±0.01cd | 0.35±0.05b |
| DS 80(55)-100(75)-RH | 9.82±0.37def | 5.78±0.31ef | 0.51±0.03cd | 2.46±0.17de | 1.11±0.07de | 1.84±0.06bc | 0.81±0.05bc | 0.43±0.02hi | 0.61±0.03b | 0.4±0.02e | 0.57±0.03g | 0.27±0.01cd |
| DS 80(65)-100(75)-RH | 10.86±0.52cde | 5.29±0.24fg | 0.53±0.02cd | 1.73±0.08g | 1.21±0.04cde | 1.96±0.08b | 0.71±0.03cde | 0.45±0.01hi | 0.55±0.01c | 0.42±0.01e | 0.53±0.02g | 0.28±0.00cd |
| DS 100(45)-80(75)-RH | 9.63±0.37defg | 4.55±0.23gh | 0.37±0.02e | 2.33±0.08ef | 1.1±0.05de | 1.45±0.08fg | 0.74±0.03cd | 0.39±0.01i | 0.46±0.02e | 0.41±0.01e | 0.56±0.02g | 0.29±0.01c |
| DS 100(55)-80(75)-RH | 10.84±0.63cde | 12.16±0.67a | 0.49±0.03cde | 4.2±0.4ab | 1.18±0.05cde | 1.59±0.06ef | 0.62±0.03ef | 0.67±0.06e | 0.99±0.05a | 0.61±0.03bc | 0.56±0.03g | 0.25±0.01de |
| DS 100(65)-80(75)-RH | 9.02±1.99fg | 7.39±1.91cd | 0.59±0.20bc | 4.45±0.87a | 1.22±0.1cd | 1.15±0.17i | 0.75±0.19cd | 0.48±0.08gh | 0.53±0.09c | 0.63±0.15b | 0.65±0.09de | 0.34±0.02b |
| F-value | 10.248 | 24.895 | 5.832 | 10.559 | 24.628 | 37.815 | 31.186 | 92.396 | 66.875 | 34.475 | 67.541 | 124.202 |
| *p*-value | <0.001 | <0.001 | <0.001 | <0.001 | <0.001 | <0.001 | <0.001 | <0.001 | <0.001 | <0.001 | <0.001 | <0.001 |

Values are expressed as mean ± standard deviation. Different superscript lowercase letters within the same column indicate significant differences ($p < 0.05$).

**Table S6** Volatile compound contents of steamed perch fish under different steaming modes.

| | Content (μg/kg) | | | | | | | | | | | |
|---|---|---|---|---|---|---|---|---|---|---|---|---|
| | Pentanal | Hexanal | Heptanal | Octanal | Nonanal | (E)-2-Octenal | Benzaldehyde | Pentanol | Heptanol | 1-Octanol | 1-Octen-3-ol | 2-Pentylfuran |
| SS 100 | 1.56±0.39ef | 24.65±3.06e | 2.56±0.03ghi | 4.12±0.55d | 10.45±0.29fgh | nd | 1.15±0.61f | 0.87±0.02d | 1.05±0.27d | 1.07±0.13c | 5.40±2.1ef | nd |
| DS 80(45)-100 | 4.16±1.00de | 66.33±7.6de | 7.01±2.00cdef | 3.45±0.42d | 8.60±1.95gh | 3.33±0.09c | 3.67±0.10d | 3.21±0.89cd | 1.49±0.13d | 3.04±0.22c | 9.24±0.13def | 3.64±0.01d |
| DS 80(55)-100 | 1.99±0.30ef | 23.90±10.16e | 1.07±0.09i | 3.10±0.44d | 4.50±0.22h | 0.51±0.14c | 2.91±0.63de | 1.17±0d | 0.65±0.14d | 0.77±0.27c | 3.96±0.51f | 0.73±0.1fg |
| DS 80(65)-100 | 3.58±0.48def | 71.82±7.99de | 3.77±0.70fghi | 4.94±0.75d | 6.29±1.02h | 1.31±0.22c | 1.66±0.25ef | 3.23±0.12cd | 1.43±0.06d | 2.40±0.86c | 10.71±0.25def | 1.28±0.17efg |
| DS 100(45)-80 | 1.43±0.28f | 24.61±9.82e | 1.66±0.19hi | 1.90±0.63d | 5.58±0.20h | 1.16±0.29c | 1.60±0.17ef | 0.90±0.04d | 0.64±0.00d | 1.11±0.28c | 5.68±1.48ef | 0.74±0.11fg |
| DS 100(55)-80 | 1.65±1.20ef | 33.08±11.61e | 1.76±0.95hi | 1.11±0.60d | 3.69±0.99h | 0.98±0.15c | 0.30±0.25f | 1.37±0.73d | 0.88±0.37d | 0.79±0.33c | 1.85±0.98f | 0.32±0.05g |
| DS 100(65)-80 | 5.31±0.65d | 65.32±16.18de | 5.56±0.03cdefg | 4.36±1.40d | 7.63±0.74gh | 2.47±0.76c | 4.23±0.30cd | 3.45±0.07cd | nd | 1.72±0.26c | 14.89±4.73cde | 3.18±0.17d |
| SS 100(75)-RH | 3.24±0.90def | 98.48±2.83d | 4.24±0.49efgh | 3.57±0.42d | 16.32±5.71ef | nd | 3.93±0.47cd | 4.28±0.49bcd | nd | 1.73±0.71c | 15.71±1.60cd | 2.74±0.58de |
| DS 80(45)-100(75)-RH | 2.01±1.15ef | 116.35±45.27d | 4.97±2.02defgh | 3.92±0.19d | 20.27±2.28e | 2.40±0.28c | 3.68±0.45d | 6.74±3.54bc | 8.08±0.11c | 8.13±1.40b | 20.80±3.14c | 2.23±0.24def |
| DS 80(55)-100(75)-RH | 15.96±0.39b | 263.08±0.13b | 7.76±1.20cde | 31.81±6.80b | 36.94±5.54c | 16.31±3.90a | 3.98±0.42cd | nd | 16.89±3.53a | 17.60±3.65a | 72.20±11.52a | 6.47±0.14c |
| DS 80(65)-100(75)-RH | 5.34±0.35d | 95.07±2.30d | 8.35±0.07cd | 7.76±0.76d | 14.07±0.55efg | 1.50±0.58c | 5.18±0.75c | 2.37±0.29d | 2.08±0.08d | 3.08±0.44c | 20.61±2.20c | 2.50±0.23de |
| DS 100(45)-80(75)-RH | 8.69±3.04c | 170.40±43.19c | 9.18±3.77c | 18.58±4.57c | 55.48±7.56b | nd | 18.16±1.29a | nd | nd | nd | 31.65±8.55b | 13.90±1.89b |
| DS 100(55)-80(75)-RH | 23.13±1.50a | 448.64±51.56a | 22.51±3.38a | 54.72±7.68a | 81.79±1.52a | 13.09±3.77b | 12.25±1.25b | 10.72±3.27a | 13.42±1.82b | 19.48±3.67a | 63.24±3.72a | 25.15±0.28a |
| DS 100(65)-80(75)-RH | 14.64±0.62b | 264.31±26.57b | 17.81±0.60b | 17.73±1.51c | 30.14±0.29d | 4.66±1.35c | nd | 7.31±0.65b | nd | 7.42±1.03b | 34.60±5.59b | 13.86±1.89b |
| F-value | 89.750 | 57.536 | 31.832 | 53.174 | 155.290 | 18.276 | 118.041 | 8.827 | 45.648 | 32.807 | 58.149 | 300.499 |
| *p*-value | <0.001 | <0.001 | <0.001 | <0.001 | <0.001 | <0.001 | <0.001 | <0.001 | <0.001 | <0.001 | <0.001 | <0.001 |

Values are expressed as mean ± standard deviation. nd = not detected. Different superscript lowercase letters within the same column indicate significant differences ($p < 0.05$).

**Table S7** Summary of one-way ANOVA results for quality indicators of steamed perch fish under different steaming modes

| **Quality index** | **F-value** | ***p*-value** | **Significance** |
|---|---|---|---|
| Color | 0.615 | 0.833 | ns |
| Completeness | 0.847 | 0.611 | ns |
| Structure | 0.972 | 0.488 | ns |
| Springiness | 0.941 | 0.517 | ns |
| Tenderness | 0.782 | 0.675 | ns |
| Chewiness | 1.340 | 0.214 | ns |
| Juiciness | 0.952 | 0.507 | ns |
| Cooked fish | 1.269 | 0.255 | ns |
| Grassy | 0.354 | 0.979 | ns |
| Fatty | 1.282 | 0.248 | ns |
| Mushroom-like | 1.156 | 0.332 | ns |
| Fishy | 3.094 | 0.001 | * |
| Umami | 1.194 | 0.307 | ns |
| Bitter | 0.701 | 0.755 | ns |
| Shear force | 11.387 | <0.001 | * |
| Hardness | 5.349 | 0.001 | * |
| Chewiness | 2.283 | 0.064 | ns |
| Cohesiveness | 1.737 | 0.153 | ns |
| Springiness | 2.061 | 0.091 | ns |
| Resilience | 1.525 | 0.216 | ns |
| Glu | 317.981 | <0.001 | * |
| IMP | 5.318 | <0.001 | * |
| AMP | 39.600 | <0.001 | * |
| EUC | 248.916 | <0.001 | * |

| | | | |
|---|---|---|---|
| Ala | 10.248 | <0.001 | * |
| Gly | 24.895 | <0.001 | * |
| Ser | 5.832 | <0.001 | * |
| His | 10.559 | <0.001 | * |
| Lys | 24.628 | <0.001 | * |
| Met | 37.815 | <0.001 | * |
| Val | 31.186 | <0.001 | * |
| Phe | 92.396 | <0.001 | * |
| Cys | 66.875 | <0.001 | * |
| Ile | 34.475 | <0.001 | * |
| Leu | 67.541 | <0.001 | * |
| Tyr | 124.202 | <0.001 | * |
| Pentanal | 89.750 | <0.001 | * |
| Hexanal | 57.536 | <0.001 | * |
| Heptanal | 31.832 | <0.001 | * |
| Octanal | 53.174 | <0.001 | * |
| Nonanal | 155.290 | <0.001 | * |
| (*E*)-2-Octenal | 18.276 | <0.001 | * |
| Benzaldehyde | 118.041 | <0.001 | * |
| Pentanol | 8.827 | <0.001 | * |
| Heptanol | 45.648 | <0.001 | * |
| 1-Octanol | 32.807 | <0.001 | * |
| 1-Octen-3-ol | 58.149 | <0.001 | * |
| 2-Pentylfuran | 300.499 | <0.001 | * |

**Table S8** The rOAV values of volatile aroma compounds in perch fish under different steaming modes.

| Compounds | Odors | Threshold (μg/100g) | rOAV | | | | | | | | | | | | | |
|---|---|---|---|---|---|---|---|---|---|---|---|---|---|---|---|---|
| | | | SS 100 | DS 80(45)-100 | DS 80(55)-100 | DS 80(65)-100 | DS 100(45)-80 | DS 100(55)-80 | DS 100(65)-80 | SS 100(75)-RH | DS 80(45)-100(75)-RH | DS 80(55)-100(75)-RH | DS 80(65)-100(75)-RH | DS 100(45)-80(75)-RH | DS 100(55)-80(75)-RH | DS 100(65)-80(75)-RH |
| Octanal | fatty/soapy | 0.05 | 8.2 | 6.9 | 6.2 | 9.9 | 3.8 | 2.2 | 8.7 | 7.1 | 7.8 | 63.6 | 14.0 | 37.2 | 109.4 | 35.5 |
| Hexanal | green/grassy | 0.5 | 5.6 | 13.3 | 4.8 | 14.4 | 4.9 | 6.6 | 13.1 | 19.7 | 23.3 | 52.6 | 19.0 | 34.1 | 89.7 | 52.9 |
| Nonanal | fatty/citrus | 0.1 | 10.4 | 8.6 | 4.1 | 6.3 | 5.6 | 3.7 | 7.6 | 16.3 | 23.0 | 36.9 | 12.5 | 55.5 | 81.8 | 29.3 |
| 1-Octen-3-ol | mushroom | 0.1 | 5.4 | 9.2 | 4.0 | 10.7 | 5.7 | 1.9 | 14.9 | 15.7 | 20.8 | 72.2 | 19.8 | 31.6 | 63.2 | 34.6 |
| Heptanal | fatty/fresh | 0.28 | 0.9 | 2.5 | 0.4 | 1.2 | 0.6 | 0.6 | 2.0 | 1.5 | 1.8 | 2.8 | 2.7 | 3.3 | 8.0 | 6.4 |
| (*E*)-2-Octenal | cucumber | 0.3 | - | 1.1 | 0.2 | 0.4 | 0.4 | 0.3 | 0.8 | - | 0.8 | 5.4 | 0.5 | - | 4.4 | 1.6 |
| 2-Pentylfuran | fruity/metallic | 0.58 | - | 0.6 | 0.1 | 0.2 | 0.1 | 0.1 | 0.5 | 0.5 | 0.5 | 1.1 | 0.4 | 2.4 | 4.3 | 2.4 |
| Pentanal | nutty | 2 | 0.1 | 0.2 | 0.1 | 0.2 | 0.1 | 0.1 | 0.2 | 0.2 | 0.1 | 0.8 | 0.3 | 0.4 | 1.2 | 0.6 |
| Heptanol | fresh/green | 0.5 | 0.2 | 0.3 | 0.1 | 0.3 | 0.1 | 0.2 | - | - | 1.6 | 3.4 | 0.4 | - | 2.7 | - |
| 1-Octanol | green/fruity | 12 | - | - | - | - | - | - | - | - | 0.1 | 0.1 | - | - | 0.2 | 0.1 |
| Pentanol | balsam | 15 | - | - | - | - | - | - | - | - | - | - | - | - | 0.1 | - |
| Benzaldehyde | almond | 75 | - | - | - | - | - | - | - | - | - | - | - | - | - | - |

The threshold was referenced from a review[1].

## References


1. Sohail, A.; Al-Dalali, S.; Wang, J.; Xie, J.; Shakoor, A.; Asimi, S.; Shah, H.; Patil, P. Aroma Compounds Identified in Cooked Meat: A Review. *Food Research International* **2022**, *157*, 111385, doi:10.1016/j.foodres.2022.111385.
